# Effect of Automated Telephone Infectious Disease Consultations to Nonacademic Hospitals on 30-Day Mortality Among Patients With *Staphylococcus aureus* Bacteremia: The SUPPORT Cluster Randomized Clinical Trial

**DOI:** 10.1001/jamanetworkopen.2022.18515

**Published:** 2022-06-24

**Authors:** Sebastian Weis, Stefan Hagel, Julia Palm, André Scherag, Steffi Kolanos, Christina Bahrs, Bettina Löffler, Roland P. H. Schmitz, Florian Rißner, Frank M. Brunkhorst, Mathias W. Pletz

**Affiliations:** 1 Institute for Infectious Diseases and Infection Control, Jena University Hospital, Friedrich-Schiller University, Jena, Germany; 2 Center for Sepsis Control and Care, Jena University Hospital, Friedrich-Schiller University, Jena, Germany; 3 Department of Anesthesiology and Intensive Care, Jena University Hospital, Friedrich-Schiller University, Jena, Germany; 4 Institute of Medical Statistics, Computer, and Data Sciences, Jena University Hospital, Friedrich-Schiller University, Jena, Germany; 5 Department of Medicine I, Division of Infectious Diseases and Tropical Medicine, Medical University of Vienna, Vienna, Austria; 6 Institute of Medical Microbiology, Jena University Hospital, Friedrich-Schiller University, Jena, Germany; 7 Center for Clinical Studies, Jena University Hospital, Friedrich-Schiller University, Jena, Germany

## Abstract

**Importance:**

*Staphylococcus aureus* bacteremia (SAB) is a common and potentially severe infectious disease (ID). Retrospective studies and derived meta-analyses suggest that bedside infectious disease consultation (IDC) for SAB is associated with improved survival; however, such IDCs might not always be possible because of the lack of ID specialists, particularly at nonacademic hospitals.

**Objectives:**

To investigate whether unsolicited telephone IDCs (triggered by an automated blood stream infection reporting system) to nonacademic hospitals improved 30-day all-cause mortality in patients with SAB.

**Design, Setting, and Participants:**

This patient-blinded, multicenter, interventional, cluster randomized, controlled, crossover clinical trial was conducted in 21 rural, nonacademic hospitals in Thuringia, Germany. From July 1, 2016, to December 31, 2018, 1029 blood culture reports were assessed for eligibility. A total of 386 patients were enrolled, whereas 643 patients were not enrolled for the following reasons: death before enrollment (n = 59); palliative care (n = 41); recurrence of SAB (n = 9); discharge from the hospital before enrollment (n = 77); age younger than 18 years (n = 5); duplicate report from a single patient (n = 26); late report (n = 17); blood culture reported during the washout phase (n = 48); and no signed informed consent for other or unknown reasons (n = 361).

**Interventions:**

During the ID intervention phase, ID specialists from Jena University Hospital provided unsolicited telephone IDCs to physicians treating patients with SAB. During the control phase, patients were treated according to local standards. Crossover was performed after including 15 patients or, at the latest, 1 year after the first patient was included.

**Main Outcomes and Measures:**

Thirty-day all-cause mortality.

**Results:**

A total of 386 patients (median [IQR] age, 75 [63-82] years; 261 [67.6%] male) were included, with 177 randomized to the IDC group and 209 to the control group. The 30-day all-cause mortality rate did not differ between the IDC and control groups (relative risk reduction [RRR], 0.12; 95% CI, −2.17 to 0.76; *P* = .81). No evidence was found of a difference in secondary outcomes, including 90-day mortality (RRR, 0.17; 95% CI, −0.59 to 0.57; *P* = .62), 90-day recurrence (RRR, 0.10; 95% CI, −2.51 to 0.89; *P* = .89), and hospital readmission (RRR, 0.04; 95% CI, −0.63 to 0.48; *P* = .90). Exploratory evidence suggested that indicators of quality of care were potentially realized more often in the IDC group than in the control group (relative quality improvement, 0.16; 95% CI, 0.08-0.26; *P* = .01).

**Conclusions and Relevance:**

In this cluster randomized clinical trial, unsolicited telephone IDC, although potentially enhancing quality of care, did not improve 30-day all-cause mortality in patients with SAB.

**Trial Registration:**

drks.de Identifier: DRKS00010135

## Key Points

**Question:**

Do unsolicited telephone infectious disease consultations for nonacademic hospitals improve 30-day all-cause mortality in patients with *Staphylococcus aureus* bacteremia?

**Findings:**

In this cluster randomized crossover clinical trial of 386 participants in 21 centers in Thuringia, Germany, no effect was found from the telephone consultations on 30-day all-cause mortality. Further explorative analyses revealed potential, modest improvements in quality-of-care indicators.

**Meaning:**

This trial found that telephone consultations did not have an effect on survival despite improvements in some quality-of-care indicators, suggesting that bedside consultations should remain the stand of care.

## Teaser

This cluster randomized clinical trial assesses whether unsolicited telephone infectious disease consultations affect mortality in patients with *Staphylococcus aureus* bacteremia at nonacademic hospitals.

## Introduction

Infectious disease consultation (IDC) is a powerful tool for antibiotic stewardship fostering optimized antimicrobial treatment and improving patient outcomes.^1,2,3,4,5,6,7^ Benefits are best documented for patients with *Staphylococcus aureus* bacteremia (SAB),^3,8,9^ for which the accepted standard of care includes intravenous antibiotic therapy of appropriate duration,^10,11^ source control, and a detailed examination for metastatic infection or endocarditis.^3,9,12^ Previous data from quasi-experimental studies showed that adherence to a bundle of evidence-based quality-of-care indicators (QIs) was associated with improved patient care and decreased mortality.^3,13,14^ However, the available evidence has been exclusively derived from retrospective studies and therefore bears a high inherent risk of bias. In addition, only 1 previous retrospective study,^14^ with 342 patients, compared bedside consultation, telephone consultation, and no consultation for SAB. The study^14^ found improved outcomes only for bedside consultation compared with no consultation, but only 62 patients with telephone consultation and 35 patients with no consultation were included, and all patients were treated at a single university hospital. In practice, most patients with SAB are hospitalized in small- or medium-sized nonacademic hospitals that might not be able to perform bedside IDC because of a shortage of infectious disease (ID) specialists, which is particularly true in Germany. Hence, unsolicited telephone consultation by an ID specialist from an outside university hospital could be an option to optimize the management of patients with SAB and improve the associated outcomes. In this study, we examine whether unsolicited telephone IDCs for patients with SAB treated at nonacademic hospitals improved 30-day all-cause mortality.

## Methods

### Study Design 

SUPPORT (Study on the Utility of a Statewide Counseling Program for Improving Outcomes of Patients With Staphylococcal Bacteremia in Thuringia) was a single-blinded, multicenter, interventional, cluster randomized, controlled clinical trial with a crossover design that included 21 nonacademic hospitals in Thuringia, Germany. We considered a cluster randomized design with nonacademic hospitals as clusters as the best feasible way to minimize possible bias from carryover effects when addressing this question. Individual patient-level randomization was deemed not feasible because the physicians receiving IDC for individual patients in the interventional group would likely have applied the same advice to patients in the control group. In addition, to ensure fair allocation of IDC to all participating centers and strengthen their motivation to participate in the study, a crossover trial with a washout period of 1 month when switching from intervention to control was chosen over a parallel design study. The study was conducted in accordance with Good Clinical Practice guidelines and the Declaration of Helsinki.^15^ The institutional review board of each recruiting center approved the protocol. Ethical approval was obtained from the Ethics Committee of Jena University and from the Ethics Committee of the State Chamber of Physicians of Thuringia. The trial protocol has been published previously.^16^ The full translated protocol and its amendments are provided in Supplement 1. All participants or their legally authorized representatives provided written informed consent before inclusion in the study. This study followed the Consolidated Standards of Reporting Trials (CONSORT) reporting guideline and its extension for cluster randomized trials.^17,18^

**Figure 1.**
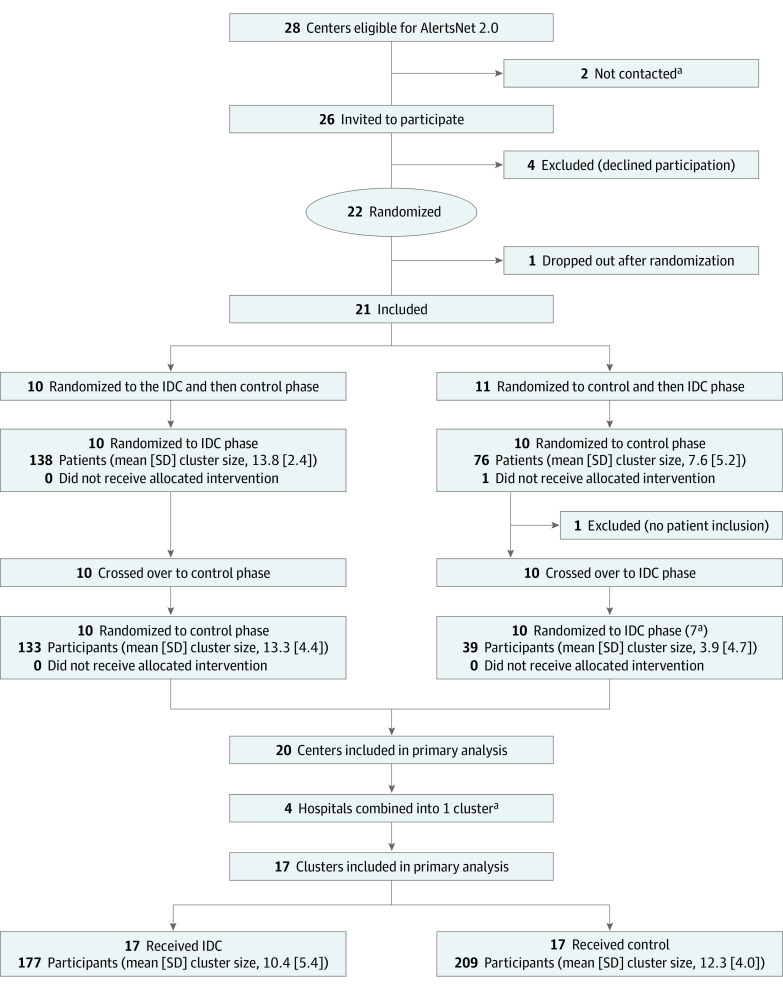
Flow Diagram of the SUPPORT Trial IDC indicates infectious disease consultation. ^a^Twenty centers underwent crossover and completed both the IDC and control phases; however, because of very low recruitment rates, 4 centers were combined to form a reasonable cluster size, resulting in 17 clusters for analysis.

Participating centers were randomized to receive the intervention phase (ICD) followed by the control phase (standard of care) or vice versa, with each center allocated to 1 of the 2 randomization sequences. Randomization was performed using block randomization with variable block length by the Center for Clinical Studies at Jena University Hospital (JUH). Crossover to the second phase occurred after 15 included patients completed the first phase or at 12 months after trial initiation at each study center. A 1-month washout period was included between phases to minimize carryover effects (Figure 1).

### Recruitment of Centers and Patients

We conducted the study in Thuringia, Germany, where a statewide automated blood culture (BC) registry, AlertsNet 2.0, was recently implemented. This registry reported pathogens after identification and testing of antimicrobial resistances central to JUH^19,20^ and allowed for statewide, real-time identification of patients with SAB.^21^ From July 1, 2016, to December 31, 2018, a total of 1029 BC reports were assessed for eligibility.

Centers were eligible for participation in the SUPPORT trial if they were part of the AlertsNet 2.0 registry.^20^ Patients were identified by an automatic daily review of microbiological BC reports that occurred as part of the AlertsNet 2.0 registry. Data management of SUPPORT was done separately. On registration of a case of SAB, a fax with anonymized information (ie, the information of a SAB detection at a specific study site and an automatic subject identification code) was sent to the study staff at JUH. At the same time, the system sent a fax to the hospital at which the patient was admitted. The system reported once the antimicrobial resistance test was available. Study centers could also include a patient with SAB if the patient was identified before the automatic reporting by AlertsNet 2.0.

After being informed, the staff of the JUH contacted the study personnel at the respective study site, informed them about the presence of a case of SAB, and requested that informed consent be obtained from the patient by trained medical staff. Only after the participant agreed and signed the consent form did the study center receive identifiable information on the participant. For patients in the intervention group, the telephone consultation with the treating physician was performed, and the consultation was subsequently confirmed by fax (including therapy proposal). For patients in the control group (no IDC), the 2 aforementioned steps were omitted (ie, after inclusion in the study, only the informed consent form was faxed to the study center, and no IDC was performed). Patients were blinded to the center allocation. All physicians were instructed to maintain patient blinding to center allocation. Additional information is given in the eMethods in Supplement 2.

### Patient Inclusion and Exclusion Criteria

All adult patients (≥18 years of age), regardless of sex, with at least 1 positive BC result with SAB who provided written consent (themselves or via their legal representative) were included. Patients who had been previously enrolled in the study were reenrolled only if the second SAB episode occurred more than 90 days after the first SAB episode and if there was no evidence of recurrence from a deep-seated infection. Patients were excluded for the following reasons: (1) BC positivity for only bacteria other than *S aureus*, (2) *S aureus* infection without bacteremia, or (3) palliative care and therapy limitations or a life expectancy of less than 90 days because of an underlying disease. A total of 386 patients were enrolled, whereas 643 patients were not enrolled for the following reasons: death before enrollment (n = 59); palliative care (n = 41); recurrence of SAB (n = 9); discharge from the hospital before enrollment (n = 77); younger than 18 years (n = 5); duplicate report from a single patient (n = 26); late report (n = 17); BE reported during the washout phase (n = 48); and no signed informed consent for other or unknown reasons (n = 361). Individual informed consent from each patient was requested by the ethics committee and was obtained by the study personnel of the local hospital (typically not the treating physicians). The treating physician was informed about the inclusion of the patients, and the contact details were provided to the ID service that performed routine SAB bedside consultations at JUH, which then initiated the telephone consultation.

### Telephone Consultation

The ID intervention consisted of an unsolicited telephone consultation by an ID specialist from JUH with the treating physician. The ID specialist recommended state-of-the-art, personalized management of SAB based on 6 QIs that had been successfully implemented at JUH^13^: (1) drawing of follow-up BC, (2) early source control or focus on sanitation, (3) transesophageal echocardiography in patients with clinical indications, (4) intravenous narrow-spectrum β-lactam antibiotics for methicillin-susceptible *S aureus*, (5) adjustment of the vancomycin dose according to the trough levels in patients with methicillin-resistant *S aureus* infections, and (6) treatment duration according to the complexity of infection (ie, 2 weeks for uncomplicated infections and at least 4 weeks for complicated infections). The IDC was initiated shortly after obtaining informed consent from the study center, typically on the same day. In case the treating physician could not be reached, the contact was reestablished the following day or, in the case of Fridays, either over the weekend if the treating physician was available or on the next workday. Consultation included detailed information about past and current medical history and the current concerns of the patient, the results of the physical examination, the presence of implanted foreign bodies, the course of serologic markers of inflammation and organ function, any clinical tests and interventions that were planned or had already been performed, and current antibiotic therapy.

After the IDC, a summary of the individual recommendations following a standardized structure was additionally provided by fax. This summary typically included the recommendation of the antibiotic of choice, such as cephazolin or flucloxacillin for methicillin-susceptible SAB and intravenous daptomycin or vancomycin with trough-level assessment for methicillin-resistant SAB, 14 days of intravenous therapy for uncomplicated SAB or longer for complicated SAB, the drawing of follow-up BC samples ideally 48 to 72 hours after initiation of adequate therapy or source control, addition of a second antibiotic (eg, rifampicin or fosfomycin) in the case of suspected or proven foreign-body infection or abscesses, and exclusion of echocardiography in high-risk patients.^3,12,22,23,24,25^ Additional support was possible on request.

### Outcomes

The primary outcome of the SUPPORT trial was 30-day all-cause mortality relative to the date of the first *S aureus*–positive BC result. The secondary outcomes were (1) adherence to 6 selected QIs, (2) 90-day all-cause mortality, (3) 90-day recurrence rate, and (4) development of secondary septic foci. An additional 18-month (long-term) survival outcome was added during the trial. We predetermined that SAB occurring after 90 days would be considered a new episode and not a recurrence.

To assess possible carryover effects, we also compared the results in relation to the sequence of the 2 study phases in a post hoc analysis. All statistical outcome tests were performed at the cluster level. We initially also intended to assess sepsis and septic shock,^16^ as defined by Singer et al^26^; however, relevant information for the assessment of sepsis parameters was missing for several patients, and because overall mortality was low, the development of sepsis could not be assessed conclusively.

### Patient Follow-up

Patients were contacted by telephone 30 days, 90 days, and 18 months after the date of the first *S aureus*–positive BC result. All data were collected by a team of specially trained rotating study nurses from JUH, who visited the study sites and reviewed patient records. The follow-up telephone interview with surviving patients was performed using a structured questionnaire that collected information regarding patients’ previous hospital admissions and complaints. If the patient could not be contacted, a relative from the same household or the general practitioner was contacted.

### Statistical Analysis

The sample size was calculated for the binary primary outcome (30-day all-cause mortality) using simulations as described previously.^27^ On the basis of a previous meta-analysis,^9^ the risk ratio between the 2 groups was estimated to range from 0.5 to 0.7 (ie, IDC may decrease 30-day all-cause mortality by up to 50%). The planned minimum of 15 centers with a mean of 2 × 15 patients each would give this study a power of greater than 80% to detect a true risk ratio of 0.5 for a control group mortality rate of 0.25 to 0.59 and moderate to low between-center variation.^16^

For the analysis of the primary and the similar secondary end points, we used the mean of the cluster-specific relative risk reduction (RRR) in percentage as the test statistic and generated the associated H_0_ distribution using 100 000 permutations. To calculate 95% CIs for further validation of these results, we used a nonparametric bootstrapping with 10 000 repetitions. This procedure was similarly applied to all analyses of the secondary end points. Sensitivity analyses followed the approach of the primary analysis and were complemented by Kaplan-Meier analyses at the individual level. We conducted 1 confirmatory test for the primary outcome such that the type I error in a strong sense was controlled at a level α = .05 (2-sided). All other analyses, including those related to secondary outcomes, are exploratory (and some are post hoc); all results of the exploratory analyses were not adjusted for multiplicity. Unless otherwise indicated, all analyses were performed according to the intention-to-treat principle. All analyses were performed in R, version 4.0.3 (R Foundation for Statistical Computing). Statistical analyses were completed in June 2021. Further information is given in the eMethods in Supplement 2.

## Results

### Participant and Center Flow Through the Trial

A total of 386 patients (median [IQR] age, 75 [63-82] years; 261 [67.6%] male and 125 [32.4%] female) were included, with 177 randomized to the IDC group and 209 to the control group (Table 1; eTables 1-3 in Supplement 2). During the study period, 26 hospitals reported data to AlertsNet 2.0. Four centers declined to participate. One center was initially randomized but then withdrew from the study. One additional hospital was initially included but was excluded before the crossover because of a lack of patient enrollment. Because 4 of the remaining 20 centers had insufficient recruitment and were combined into 1 cluster for analysis, a total of 17 clusters were included in the primary analysis (Figure 1). The mean (SD) cluster size was 12.3 (4.0) patients in the control group and 10.4 (5.4) patients in the IDC group. Notably, 2 major centers had established antibiotic stewardship services early during the study, including mandatory bedside consultations for all patients with SAB. Therefore, we conducted an additional sensitivity analysis without these 2 centers.

**Table 1.** Patient-Level Characteristics (Not Cluster Adjusted)^a^

Characteristic	All patients (n = 386)	IDC group (n = 177)	Control group (n = 209)
Age, median (IQR), y	75 (63-82)	71 (61-81)	77 (65-82)
Sex			
Male	261 (67.6)	115 (65.0)	146 (69.9)
Female	125 (32.4)	62 (35.0)	63 (30.1)
Antimicrobial resistance			
Oxacillin-methicillin	21 (5.4)	14 (7.9)	7 (3.3)
Rifampicin	0	0	0
Fosfomycin	3 (0.8)	3 (1.7)	0
Daptomycin	0	0	0
Fluoroquinolones	62 (16.1)	31 (17.5)	31 (14.8)
Linezolid	0	0	0
Missing *Staphylococcus aureus* resistance information	5 (1.3)	3 (1.7)	2 (1.0)
Implant			
Hip prosthesis	28 (7.3)	11 (6.2)	17 (8.1)
Knee prosthesis	27 (7.0)	8 (4.5)	19 (9.1)
Cardiac valve prosthesis	1 (0.3)	0	1 (0.5)
Pacemaker	51 (13.2)	24 (13.6)	27 (12.9)
Implantable cardioverter defibrillator	20 (5.2)	14 (7.9)	6 (2.9)
Vascular catheters			
Central venous catheter	31 (8.0)	15 (8.5)	16 (7.7)
Shaldon, PICC, or tunneled	36 (9.3)	22 (12.4)	14 (6.7)
Port	28 (7.3)	13 (7.3)	15 (7.2)
Mode of acquisition^b^			
Community acquired^b^	151 (39.1)	72 (40.7)	79 (37.8)
Health care system associated^c^	39 (10.1)	18 (10.2)	21 (10.0)
Nosocomial^d^	196 (50.8)	87 (49.2)	109 (52.2)
Infection focus			
Intrathoracic	66 (17.1)	39 (22.0)	27 (12.9)
Urogenital or renal	55 (14.2)	24 (13.6)	31 (14.8)
Central nervous system	5 (1.3)	2 (1.1)	3 (1.4)
Bone or joint	85 (22.0)	36 (20.3)	49 (23.4)
Cardiovascular	20 (5.2)	6 (3.4)	14 (6.7)
Otolaryngology	1 (0.3)	1 (0.6)	0
Intra-abdominal	17 (4.4)	7 (4.0)	10 (4.8)
Skin or soft tissue	159 (41.2)	75 (42.4)	84 (40.2)
Postsurgical wound infection	41 (10.6)	13 (7.3)	28 (13.4)
Peripheral catheter suspected as focus	41 (10.6)	26 (14.7)	15 (7.2)
Other catheter-related infection	63 (16.3)	31 (17.5)	32 (15.3)
Pitt score, median (IQR)	1 (0-1)	1 (0-1)	1 (0-2)
Charlson Comorbidity Score, median (IQR)	3 (1-4)	3 (1-4)	3 (1-4)
Polymicrobial infection	14 (3.6)	6 (3.4)	8 (3.8)
SAB severity			
Uncomplicated	180 (46.6)	87 (49.2)	93 (44.5)
Complicated	206 (53.4)	90 (50.8)	116 (55.5)
Endocarditis or septic metastasis^e^	83 (21.5)	40 (22.6)	43 (20.6)
Endoprosthesis^f^	317 (82.1)	143 (80.8)	174 (83.3)
Follow-up blood culture data available	179 (46.4)	96 (54.2)	83 (39.7)
Positive follow-up blood culture on days 2-4^g^	66 (17.1)	33 (18.6)	33 (15.8)
Fever within 72 h after therapy initiation^h^	74 (19.2)	30 (16.9)	44 (21.1)
Remaining catheter in patients with catheter-related infection^i^	22 (5.7)	9 (5.1)	13 (6.2)
Length of hospital stay, median (IQR), d	21 (15-29)	21 (16-30)	21 (14-29)
Time from first BC to IDC, median (IQR)^j^	NA	5 (4-7)	NA

Abbreviations: BC, blood culture; IDC, infectious disease consultation (via telephone); NA, not applicable; PICC, peripherally inserted central catheter; SAB, *Staphylococcus aureus* bacteremia.

^a^ Data are presented as number (percentage) of patients unless otherwise indicated.

^b^ Community acquired indicates signs of infection are present that are judged to have begun before hospital or less than 48 hours after the start of hospitalization (without criteria for health care system–associated SAB).

^c^ Health care system associated indicates in-hospital presentation less than 48 hours after admission and infusion therapy, wound care, or close care by nurse or family member (within 30 days before SAB infection) or outpatient presentation to a hospital or hemodialysis practice or receipt of intravenous chemotherapy within 30 days before SAB or continuous intravenous medication at home or placement in a nursing home or stay in an acute care hospital for at least 1 day within 90 days before bloodstream infection.

^d^ Nosocomial indicates no evidence (>48 hours after admission to the hospital) that the infection was present or was in the incubation phase before admission to the hospital.

^e^ Data missing for 75 patients.

^f^ Data missing for 2 patients.

^g^ Data missing for 207 patients.

^h^ Data missing for 34 patients.

^i^ Data missing for 271 patients.

^j^ Data missing for 1 patient.

### Primary Outcome

**Figure 2.**
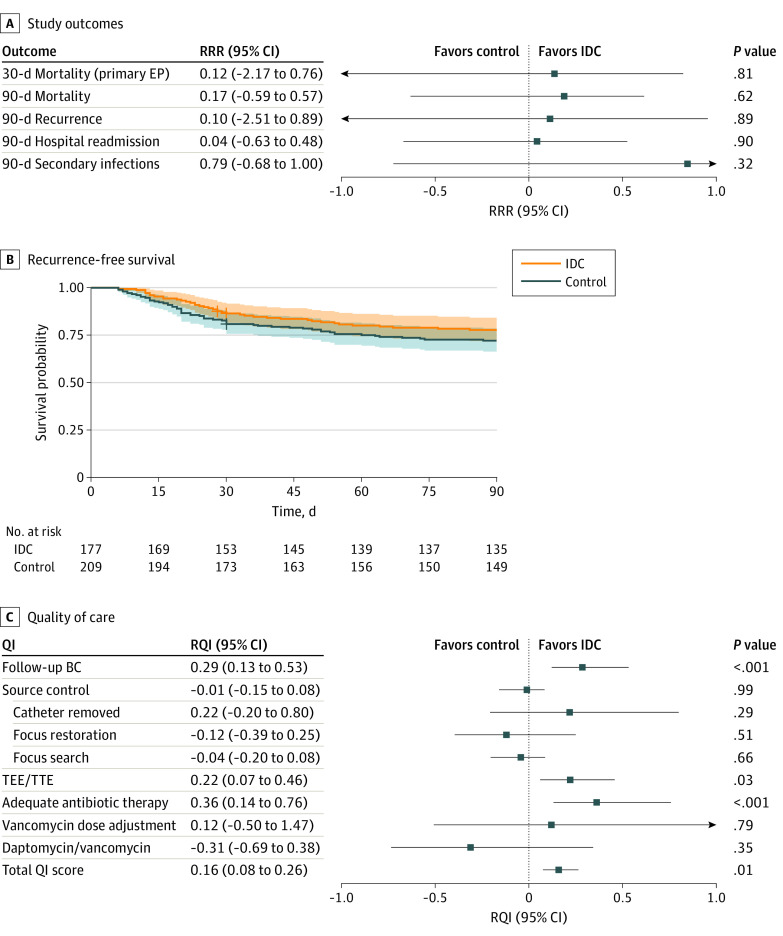
Study Outcomes by Treatment Group A, Primary and secondary outcomes. B, Kaplan-Meier plot of the composite end point (EP) of recurrence-free survival (exploratory evidence because the CIs may be biased because of the clustering of the data). C, Quality-of-care indicators (QIs). BC indicates blood culture; IDC, infectious disease consultation; RQI, relative QI improvement; RRR, relative risk reduction; TEE, transesophageal echocardiography; and TTE, transthoracic echocardiography.

The primary outcome of SUPPORT was 30-day all-cause mortality. By day 30 after the first *S aureus*–positive BC result, 7 of 177 patients (4.0%) in the IDC group and 10 of 209 patients (4.8%) in the control group had died. Thirty-eight participants in the control group and 29 participants in the IDC group were lost to follow-up. Overall, there was no evidence of a difference between the groups in the primary outcome (RRR, 0.12; 95% CI, −2.17 to 0.76; *P* = .81) at the cluster level (Figure 2A and B).

### Secondary Outcomes

By day 90, a total of 18 of 177 patients (10.2%) in the IDC group and 25 of 209 patients (12.0%) in the control group had died. Thirty-three participants in the control group and an additional 27 participants in the IDC group were lost to follow-up. Consequently, the 90-day mortality rate (RRR, 0.17; 95% CI, −0.59 to 0.57; *P* = .62), 90-day recurrence rate (RRR, 0.10; 95% CI, −2.51 to 0.89; *P* = .89), and 90-day readmission rate (RRR, 0.04; 95% CI, −0.63 to 0.48; *P* = .90) indicated no difference between groups. Exploration revealed that the predefined QIs^3^ were significantly more often realized in the IDC group (relative quality improvement [RQI], 0.16; 95% CI, 0.08-0.26; *P* = .01) (Figure 2C). Specifically, the rates of follow-up BC (RQI, 0.29; 95% CI, 0.13 to 0.53; *P* < .001), echocardiography (RQI, 0.22; 95% CI, 0.07-0.46; *P* = .03), and appropriate antibiotic therapy (RQI, 0.36; 95% CI, 0.14-0.76; *P* = .01) (Figure 2C) seemed to be higher in the IDC group.

**Figure 3.**
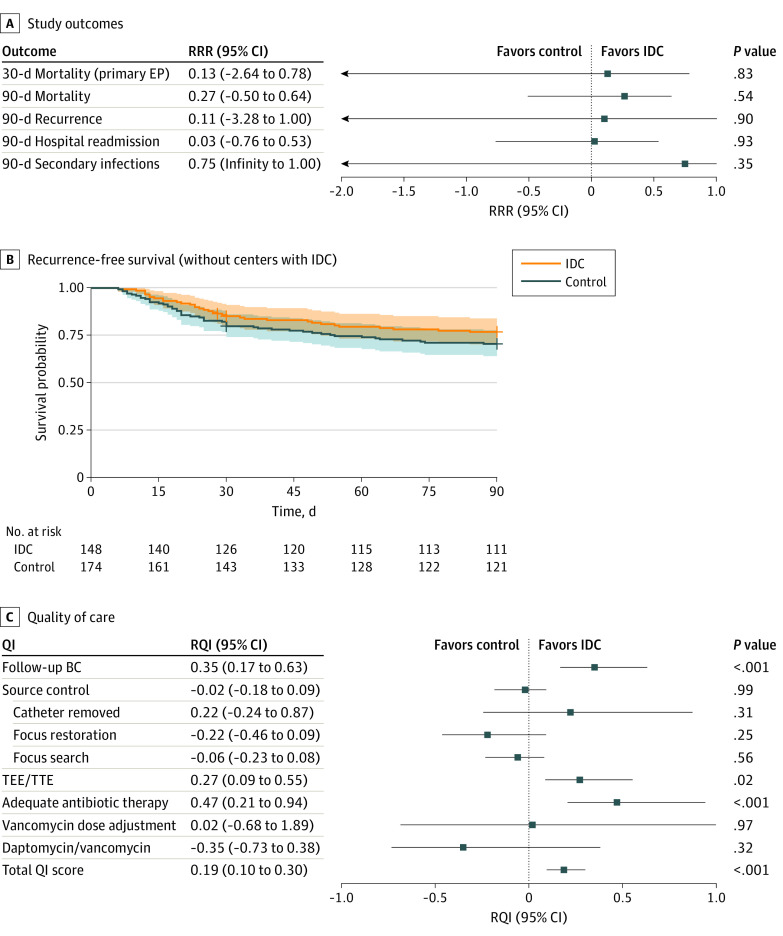
Study Outcomes by Treatment Group According to the Sensitivity Analysis Excluding the 2 Centers That Initiated Infectious Disease Consultation (IDC) Services A, Primary and secondary outcomes. B, Kaplan-Meier plot of the composite end point (EP) of recurrence-free survival (exploratory evidence because the CIs may be biased because of the clustering of the data). C, Quality-of-care indicators (QIs). BC indicates blood culture; RQI, relative QI improvement; RRR, relative risk reduction; TEE, transesophageal echocardiography; and TTE, transthoracic echocardiography.

For ethical reasons, the 2 centers that had initiated antibiotic stewardship services with bedside consultations for all patients with SAB were not prevented from providing these services during the study.^28,29^ When these 2 centers were excluded in a post hoc analysis, we obtained results similar to the primary analysis results for unsolicited telephone IDC in the 30-day mortality (RRR, 0.13; 95% CI, −2.64 to 0.78; *P* = .83) and all the secondary outcome efficacy outcomes (Figure 3A and B). The QIs also seemed to be more frequently achieved under the IDC conditions after exclusion of the 2 centers (RQI, 0.19; 95% CI, 0.10-0.30; *P* < .001) (Figure 3C).

### Exploratory Analysis

#### Adherence to Recommendations

Although follow-up bedside consultation can help reinforce recommendations to the treating physician from the ID specialist, we were interested in the extent to which the recommendations were followed after the telephone IDC. Table 2 lists the recommended and actual procedures on a patient level. Low-effort recommendations for follow-up BC (139 of 163 [85.3%]), search for foci (110 of 122 [90.2%]), narrow-spectrum antibiotic therapy (153 of 171 [89.5%]), and combination therapy (74 of 92 [80.4%]) were followed in more than 80% of the cases, whereas catheter removal (27 of 35 [77.1%]), surgical source control (26 of 39 [66.7%]), and echocardiography (102 of 148 [68.9%] with only transesophageal echocardiography performed and 61 of 148 [41.2%] with only transthoracic echocardiography performed) were less frequently performed after recommendation.

**Table 2.** Exploratory Analysis of Adherence With Recommendations in Patients Who Received Infectious Disease Consultation as Assessed by Patient Medical Records

Recommendation	No./total No. (%) of patients (N = 177)
Follow-up blood culture	139/163 (85.3)
Catheter removal	27/35 (77.1)
Source control^a^	26/39 (66.7)
Further focus search	110/122 (90.2)
TEE/TTE recommended and TEE performed	102/148 (68.9)
TEE/TTE recommended but only TTE performed	61/148 (41.2)
Narrow-spectrum antibiotic therapy^a^	153/171 (89.5)
Vancomycin dose adjustment	6/6 (100)
Alternative antibiotic therapy	70/80 (87.5)
Combination antibiotic therapy	74/92 (80.4)

Abbreviations: TEE, transesophageal echocardiography; TTE, transthoracic echocardiography.

^a^ Because of the trial-inherent delay in reporting, inclusion, and infectious disease consultation, early source control (within 72 hours) was impossible to ascertain through infectious disease consultation, and early switch to narrow-spectrum antibiotic therapy was not specifically assessed.

#### Treatment-Related Adverse Events

We observed no evidence of a group difference in the number of treatment-related adverse events (RRR, 0.20; 95% CI, −0.54 to 0.65; *P* = .59). The most frequent adverse event was an allergic reaction to antibiotic therapy (14 cases) followed by *Clostridioides difficile* enteritis (10 cases) (eTable 4 in Supplement 2).

#### Influence of the Order of Study Phases 

In a post hoc analysis, the IDC intervention seemed to have an effect when performed after the control phase than when performed before the control phase (eTable 5 in Supplement 2), suggesting that even the 1-month washout phase did not completely prevent carryover effects. This finding concerned the drawing of follow-up BCs (RQI, 0.46; 95% CI, −0.19 to 0.90; *P* = .02), exclusion of endocarditis by echocardiography (RQI, 0.58; 95% CI, 0.20-1.34; *P* = .02), and choice of an adequate antibiotic therapy (RQI, 0.68; 95% CI, 0.22-1.88; *P* = .02). Because of the limited number of events, mortality and recurrence were not assessed in this analysis.

#### Assessment of 18-Month Long-term Survival 

We also assessed long-term survival after 18 months in an exploratory post hoc analysis. Of 177 participants in the IDC group, 25 further participants were lost to follow-up, and 63 participants died. Of 209 participants in the control group, an additional 37 participants were lost to follow-up, and 71 participants died. Kaplan-Meier survival plots for this assessment are shown in the eFigure in Supplement 2. The data suggest no evidence of a difference in long-term survival between groups.

## Discussion

The multicenter SUPPORT trial investigated the effect of telephone IDC for patients with SAB in nonacademic hospitals that lack their own ID service. In this trial, IDC by telephone did not reduce the 30-day mortality rate when compared with the control group. Regarding the planned secondary end points, we detected no difference in the 90-day mortality rate between the control and IDC groups but found some evidence of improved adherence to QIs in the IDC group. Because of the potential presence of a carryover effect, the establishment of antibiotic stewardship teams in some centers, and a much lower mortality than expected, our results need to be interpreted with caution and must not be interpreted as argument against mandatory IDC for patients with SAB.

### Limitations

Our study has several limitations. First, the observed number of deaths was considerably lower than expected. Therefore, the primary sample size calculation appears to have resulted in too few patients and centers to detect 30-day mortality differences, which may have resulted from an inclusion bias toward less severe cases in non–study-experienced centers. Furthermore, there was a median of 5 days between the BC sample draw and the telephone IDC. Fifty-nine of the screened patients died before inclusion, corresponding to 9% of the nonincluded patients, which raises the question of whether an earlier intervention would have improved the outcomes for some patients. An earlier intervention, however, was not possible because of the reporting of only finalized microbiological results (ie, microscopy and other intermediate results without antibiotic resistance testing were not reported) in AlertsNet 2.0 and because of the delay in obtaining informed consent in nonexperienced centers.

Second, ID recommendations were only partially followed, mainly for low-effort measures, such as adjustment of antibiotic treatment or follow-up BCs. In a previous study,^13^ this lack of adherence was associated with fewer survival benefits than when adherence with the IDC was high. Potentially, adherence could be improved by a less anonymous ID intervention at the site, video conferencing instead of telephone conferencing, or a checklist-based report from the treating physician. These issues could be addressed in further studies.

Third, physicians’ increased awareness of SAB diagnostic and therapeutic standards attributable to participation in the study may have improved overall treatment quality also in the control group (Hawthorne effect).^30^ To convince the centers of the value of study participation, it was necessary to introduce the details of SAB management. In addition, 2 hospitals implemented antibiotic stewardship programs. We addressed this issue by excluding these 2 centers from the sensitivity analysis but found no change in the overall conclusions. For ethical reasons, we did not prevent implementation of this service in the study centers, given the important effect that ID services have on patient care and antimicrobial consumption.^28,29^

Fourth, although the number of centers was larger than planned in the protocol, we did not recruit a mean of 30 patients per center as anticipated. In fact, the numbers of patients from different centers varied more than expected, and the inclusion rate in some centers was much lower than expected. Obtaining informed consent in time was a major hurdle in nonacademic centers with limited infrastructure to support clinical trials and resulted in an above-average dropout rate. As we relied on the AlertsNet 2.0 system structure to identify potential participants, we could not increase the center numbers. In addition, increasing the recruitment time was not an option because larger centers had already finished recruiting, whereas smaller centers would have required several years to include 30 participants given the dropout rate.

## Conclusions

In summary, this cluster randomized clinical trial revealed that unsolicited telephone IDC had no effect on 30-day mortality in nonacademic hospitals. However, this intervention may increase the adherence to established QIs, as observed in exploratory analyses. Although some obstacles that may have decreased the efficacy of telephone IDC in our study (eg, the relatively long interval between BC sampling and IDC or adherence) are solvable, this study also confirms that telephone IDC is not an equivalent substitute for bedside IDC in patients with SAB. Thus, bedside IDC for patients with SAB should remain the standard of care.

## Notes

Supplement 1.
Trial Protocol

Supplement 2. **eMethods.** Supplementary Methods **eTable 1.** Patient Characteristics at the Cluster Level **eTable 2.** Additional Patient Characteristics at the Patient Level (Not Cluster Adjusted) **eTable 3.** Additional Patient Characteristics at the Cluster Level **eTable 4.** Treatment-Related Adverse Events Reported at the Patient Level (Not Cluster Adjusted) **eTable 5.** Exploratory Analysis of the Quality-of-Care Indicators Depending on the Order of Study **eFigure.** Exploratory Analysis of 18-Month Survival With Kaplan-Meier Plots for Individual Participants From (A) All Centers, (B) Centers Without Infectious Disease Consultation (IDC), and (C) for the Composite (Survival and No Readmission) for All Centers

Supplement 3.
Nonauthor Collaborators

Supplement 4.
Data Sharing Statement
