# Supplementary material for: Effect of Automated Telephone Infectious Disease Consultations to Nonacademic Hospitals on 30-Day Mortality Among Patients With Staphylococcus aureus Bacteremia: The SUPPORT Cluster Randomized Clinical Trial
Source: JAMA Netw Open. 2022 Jun 24;5(6):e2218515. doi: 10.1001/jamanetworkopen.2022.18515 (PMC9233240; doi:10.1001/jamanetworkopen.2022.18515)
Supplement: Supplement 1. — Trial Protocol [file jamanetwopen-e2218515-s001.pdf]

Study protocol

SUPPORT

Study of the utility of a statewide counseling program to improve outcomes in patients with *Staphylococcus aureus bacteremia* (SUPPORT study) -.

A single-blind, cluster-randomized trial in a cross-over design.

Study acronym:	SUPPORT
Protocol version, date:	Version 1.09 from 25.07.2016
Confidentiality Notice:	The contents of this study protocol are to be kept strictly confidential and may not be disclosed to uninvolved parties, either orally or in writing, without the consent of the study director.

Content

1 General information 4

1.1 Persons, institutions and bodies involved 4
1.2 Signatures 5
1.3 Summary 5
1.4 Synopsis 6
1.5 Schedule 7
1.6 Flow chart 8
1.7 List of abbreviations 8

2 Question or background 9

2.1 Initial situation 9
2.2 Question and justification of the project 9
2.3 Justification of treatment and examination procedures 9

3 Study objectives 10

3.1 Primary study objective and primary endpoint 10
3.2 Secondary endpoints 10

4 Study design and description 10

4.1 Type of study 10
4.2 Type of therapy assignment 10
4.3 Number and type of comparison groups 10
4.4 Scope of the study 11
4.5 Patient recruitment 11
4.6 Schedule 11

5 Participating study centers 12

5.1 List of test centers 13
5.2 Conditions for participation in the study 13

6 Selection of patients 14

6.1 Inclusion criteria 14
6.2 Exclusion criteria 14

7 Course of studies 14

7.1 Detailed study procedure 14
7.2 Patient education and consent 16
7.3 Randomization 16
7.4 Treatment phase 17
7.5 Description of laboratory and other investigations and methods 17
7.6 Examinations in the context of discharge 17
7.7 Follow-up surveys 17
7.8 Description of the individual visits 17
7.9 End of study participation 17
7.10 Premature withdrawal of a patient from the study (discontinuation criteria) 17
7.11 End of the study 18

7.11.1 Regular end of study ... 18
7.11.2 Premature end of study/discontinuation of the entire study ... 18
8 Adverse events/complications ... 18
8.1 Adverse events ... 18
8.2 Possible complications and/or risks ... 18
8.3 Recording and documentation of complications ... 18
9 Biometrics ... 19
9.1 Endpoints ... 19
9.2 Definition of evaluation collectives ... 19
9.3 Planning the scope of the study (case number planning) ... 19
9.4 Interim evaluations ... 20
9.5 Statistical analyses ... 20
9.6 Presentation of the results ... 20
10 Data management ... 20
10.1 Patient identification list ... 20
10.2 List of responsibilities ... 21
10.3 Data collection/documentation forms ... 21
10.3.1 Data collection ... 21
10.3.2 Documentation sheets ... 21
10.4 Data acquisition and processing ... 23
10.5 Study documents and their storage (archiving) ... 24
10.6 Data protection ... 25
11 Quality assurance ... 25
11.1 Standardization ... 25
11.2 Control of the study process and data quality ... 25
11.3 Monitoring data ... 25
11.4 Audits ... 25
12 Ethical concerns, legal and administrative regulations ... 26
12.1 Declaration of Helsinki and Good Clinical Practice ... 26
12.2 Ethics committees ... 26
12.3 Subsequent changes ... 26
12.4 Statutory regulations ... 26
12.5 Patient insurance ... 26
12.6 Registration ... 27
12.7 Funding ... 27
12.8 Final report and publication ... 27
13 Literature ... 28
14 Attachments ... 30
14.1 List of participating hospitals ... 30
14.2 Data protection aspects ... 31

1 General information

1.1 Persons, institutions and bodies involved

Director of Studies<br>Prof. Dr. med. **Mathias Pletz**<br>Center for Infectious Medicine and Hospital Hygiene,<br>Jena University Hospital<br>Am Klinikum 1, 07747 Jena	Deputy study director / coordinating study physician<br>**Sebastian Weis,** MD<br>Center for Infectious Medicine and Hospital Hygiene,<br>Jena University Hospital<br>Am Klinikum 1, 07747 Jena
Biometrician<br>Prof. Dr. **André Scherag**<br>Integrated Research and Treatment Center (IFB) Center for Sepsis Control and Care (CSCC).<br>Clinical epidemiology<br>Jena University Hospital<br>Am Klinikum 1, 07747 Jena	Project Management<br>Dr. rer. nat. **Roland Schmitz**<br>Integrated research and Treatment Center (IFB) Center for Sepsis Control and Care (CSCC).<br>Jena University Hospital<br>Postal address: Am Klinikum 1, 07747 Jena<br>Visitor address: Salvador-Allende-Platz 27, 07747 Jena<br><br>M.Sc. **Carolin Weber**<br>Jena University Hospital<br>Center for Sepsis Control and Care (CSCC)<br>Am Klinikum 1, 07747 Jena
Study Assistance<br>B.Sc. **Steffi Kolanos**<br>Integrated Research and Treatment Center (IFB) Center for Sepsis Control and Care (CSCC).<br>Am Klinikum 1, 07747 Jena<br>Jena University Hospital	IT Management<br>**Florian Rißner**<br>Center for Clinical Studies<br>Jena University Hospital<br>Postal address: Am Klinikum 1, 07747 Jena<br>Visitor address: Salvador Allende Square 27, 07747 Jena
Data Management<br>**Cornelia Eichhorn**<br>Center for Clinical Studies<br>Jena University Hospital<br>Postal address: Am Klinikum 1, 07747 Jena<br>Visitor address: Salvador Allende Square 27, 07747 Jena	

1.2 Signatures

Prof. Dr.med. ***Mathias Pletz***, Head of the study Date

Sebastian Weis, *M.D.*, Deputy Study Director Date

Prof. Dr.med. ***André Scherag**, Biometrician* Date

1.3 Summary

Staphylococcus aureus bacteremias (SAB) are major contributors to morbidity, mortality, and consequently healthcare costs (*1,2*). The SAB incidence in the Western world is approximately 26 per 100,000 population (*3*). About 2/3 of these infections are nosocomial-primarily catheter-associated-and about 1/3 are ambulatory-acquired (*4-6*). The 30-day mortality for nosocomial SAB was reported to be 22% in one study and 26% for ambulatory-acquired (*4*). Initial unpublished data from the AlertsNet project conducted at Jena University Hospital show a hospital mortality rate of 25%. Adequate treatment of SAB is nontrivial and includes proper choice and duration of antimicrobial therapy, infectious focus, and search for septic metastases and exclusion of infective endocarditis (*1, 7*). Data from nonrandomized and retrospective studies demonstrate that consultative co-management by clinical infectious disease specialists results in significantly improved survival, shorter hospital stays, and lower rates of complications, as well as reduced overall antibiotic use (*8-17*).

This is countered by the fact that a significant number of patients are treated in small and medium-sized hospitals. Due to the current shortage of clinical infectiologists, these cannot be cared for directly by one. In Thuringia, with more than 20,000 hospital beds, there are currently only 3 infectious disease specialist working in inpatient services. The requested cluster-randomized controlled cross-over SUPPORT study aims to test whether a telephone consultation by a clinical i infectious disease specialist increases the survival of patients with SAB in Thuringia.

142 ## 1.4 Synopsis

**Study title**	Study of the Benefits of a Nationwide Outreach Program to Improve Outcomes in Patients with *Staphylococcus aureus Bacteremia* (SUPPORT Study) - a Single-Blind, Cluster-Randomized, Cross-Over Design Study.
**Short name of the study (acronym)**	SUPPORT
**Director of Studies**	Prof. Dr.med. **Mathias Pletz**<br><br>Sepsis and Sepsis Consequences Integrated Research and Treatment Center (CSCC).<br>Center for Infectious Medicine and Hospital Hygiene<br>Jena University Hospital<br>At the clinic 1<br>07747 Jena<br>Phone: +49 (0) 3641-9324650<br>Fax: +49 (0) 3641-9324652<br>e-mail: mathias.pletz@med.uni-jena.de
**Deputy of the director of studies**	**Sebastian Weis,** MD<br><br>Sepsis and Sepsis Consequences Integrated Research and Treatment Center (CSCC).<br>Center for Infectious Medicine and Hospital Hygiene<br>Jena University Hospital<br>At the clinic 1<br>07747 Jena<br>Phone: +49 (0) 3641-9324668<br>Fax: +49 (0) 3641-9324652<br>e-mail: sebastian.weis@med.uni-jena.de
**Target population/disease**	Patients with *Staphylococcus aureus* bacteremia.
**Study design/methodology**	Prospective randomized, single-blind, controlled two-arm (intervention vs. control) multicenter trial with cross-over design. The intervention is the provision of a non-requested infectious disease telephone consult. The control group will not receive a telephone consult. The study duration in one group before cross-over is to be 12 months.<br>Randomization: 1:1 cross-over<br>Blinding: simple, i.e., patients are not told which group (intervention or control) they are in; blinding of treating physicians/study centers is not possible.<br>Intervention group: non-requested telephone consult.<br>Control group: no telephone consultation, follow-up only.<br>Follow-up: 90 days
**Aims of the clinical trial/objectives**	<u>Primary Objective:</u><br>To demonstrate efficacy (30-day all-cause mortality) and safety of nonrequested infectious disease consults compared with standard care without telephone consults in patients with SAB.

**Target variables/criteria/endpoints**	Primary outcome measure: 30-day all-cause mortality (based on time of patient's SAB diagnosis).<br><br>Secondary targets:<br>– Fulfillment of the 6 defined quality indicators of SAB treatment until hospital discharge.<br>– 90-day all-cause mortality<br>– 90-day recurrence rate<br>– Progression of SAB to sepsis/septic shock over a 90-day period.<br>– Development of septic metastases over a period of 90 days
**Number of patients**	Evaluated for study inclusion: n=550<br>Patients included: n=500<br>Patients to be analyzed (minimum): n=450<br>(15 centers with an average of 2 (for the two study phases) x 15 patients).
**Inclusion criteria**	All patients ≥ 18 years with 1st diagnosed SAB (positive blood culture). Re-inclusion if 2nd episode occurs > 90 days after 1st episode and there is no evidence of recurrence due to deep-seated infection.
**Exclusion criteria**	– Positive blood cultures with only other pathogens in the blood culture<br>– *Staphylococcus aureus* infections without bacteremia<br>– Patients with therapy limitations or cessation, palliative care.<br>– Patients with a life expectancy < 90 days
**Treatments/procedures, treatment plan (incl. aftercare).**	One-time performance of a non-requested case-based infectious disease consil.
**Schedule**	Patient-Related:<br>Duration of study-related measures: Follow-up duration: 90 days. Expected completion of last follow-up: April 30, 2018.<br><br>Study Related:<br>Start (date): August 1, 2015<br>Recruitment period: 24 months.<br>Inclusion 1st patient until end of follow-up last patient: 27 months<br><br>Total duration: 36 months.<br>Estimated completion date including evaluation: July 31, 2018.
**Study Centers**	Planned: n = 15 (participating hospitals of the AlertsNet project), listing in the appendix.
**Statistical methods**	Comparison of 30-day mortality between the intervention and control groups using a generalized mixed linear model (see the main body of the protocol for details). Null hypothesis testing was performed for the fixed effect *group* using the Wald test statistic with a two-sided significance level α=5%. Case number planning was done using simulations (see main body of the protocol for details) such that a power of 80% can be achieved to detect moderate to strong effects.
**Funding**	Federal Ministry of Education and Research (BMBF)<br>Funding code: FKZ 01EO1502

1.5 Schedule

In this regard, we refer to the schedule in **Chapter 4.6.**

1.6 Flowchart m

In this regard, we refer to the study schedule in **Chapter 7**.

1.7 List of abbreviations

CDDS	Clinical Data Document System
EBCR	Electronic Blood Culture Registry
CRF	Case Report Form, Survey Form (paper-based or electronic).
FPI	First Patient In
GCP/GCP	Good Clinical Practice, Good Clinical Practice
GLMM	Generalized Linear Mixed Model
ITT	Intention-to-Treat
LPO	Last Patient Out
MDDS	Medical Data Document System
MSSA	Methicillin-sensitive *Staphylococcus aureus*
MRSA	Methicillin-resistant *Staphylococcus aureus*
PPT	Per Protocol Analysis
SAB	*Staphylococcus aureus* bacteremia
SIRS	Systemic Inflammatory Response Syndrome
SOP	Standard Operating Procedure
UKJ	Jena University Hospital

2 Question or background

2.1 Initial situation

Bacteremias caused by methicillin-sensitive (MSSA) and methicillin-resistant (MRSA) *Staphylococcus aureus* strains are often complicated infectious diseases associated with high lethality and represent a separate entity due to the peculiarities in course, complications, and therapy (*18*). *Staphylococcus aureus* bacteremias (SAB) are relatively common, with an incidence of 22-32/100,000 (*3*) per year, and have a high lethality of up to 40% compared with other bacteremias (*17, 19-21*).

2.2 Question and justification of the project

The present study aims to answer the question to what extent non-requested telephone consultations by a clinical infectious disease specialist improve survival of patients with SAB in Thuringia.

2.3 Justification of the treatment and examination procedures

Data from nonrandomized and retrospective studies demonstrate that the inclusion of a clinical infectious disease specialist significantly improves the treatment and survival of SAB patients. Patients seen in consultation by an infectious disease specialist had higher rates of echocardiography, adequate duration of antibiotic therapy, and subsequently improved 30-day survival despite having more initial risk factors (*8,9,17*). A separate meta-analysis of 16 studies demonstrated that infectious disease consults can lead to a halving of SAB mortality (13% vs. 27%) (*22*). A recent study also demonstrated that adherence to an evidence-based bundle of measures improves the management of patients with SAB and reduces 14- and 30-day mortality (*11*). In contrast, a significant number of patients are treated in small and medium-sized hospitals and, because of the current shortage of clinical infectious disease specialists, cannot be directly managed by one. In Thuringia, with more than 20,000 hospital beds, there are currently only 3 clinical infectious disease specialist working in inpatient services.

The study aims to assess the extent to which non-requested telephone consultations in non-university hospitals in Thuringia significantly improve survival of patients with *Staphylococcus aureus* bacteremia. An overview of the procedure in the therapy arms is given in **section 7.1.** It is estimated that approximately 250 patients per year can be included in the study. If the study objectives can be confirmed by the results, it is conceivable that infectious disease telephone consults will be defined as the standard of care for all patients with SAB.

3 Study objectives

3.1 Primary study objective and primary endpoint

The primary study objective of the SUPPORT study is to determine whether 30-day all-cause mortality can be reduced in patients with SAB using telephone consults with a clinical infectious disease specialist in Thuringia (compared to no such telephone consults). This is a superiority study.

Primary endpoint: 30-day all-cause mortality

3.2 Secondary endpoints

The secondary endpoints of the SUPPORT study are:

- Fulfillment of the 6 defined quality indicators
- 90-day all-cause mortality
- 90-day recurrence rate
- Antibiotic therapy performed
- Progression of SAB to sepsis/septic shock over a 90-day period.
- Development of septic metastases over a period of 90 days

4 Study design and description

4.1 Type of study

SUPPORT is a controlled, cluster-randomized, single-blind, prospective cross-over study.

4.2 Type of therapy assignment

Participating hospitals will be randomized into two sequences before the start of the study. The randomization ratio for the two study arms is 1:1. It is intended to blind patients, i.e., no patient learns whether or not his or her treating physician receives a telephone consultation. Blinding of study physicians is not possible. The change from the intervention group to the control group or from the control group to the intervention group (cross-over) will take place for each hospital individually after inclusion of the 17th patient or after one year at the latest. The aim is to include a total of 30 patients per hospital.

4.3 Number and type of comparison groups

Two parallel groups will be formed. The first group represents the intervention group, in which randomized hospitals receive a non-requested telephone consult by a clinical infectious disease specialist when SAB is detected. The second group represents the control group, in which SAB is recorded and patients are followed up, but in which no nonrequested telephone consultation by a clinical infectious disease specialist occurs when SAB is detected. After inclusion of 250 patients, but no later than 12 months after initiation of inclusion, *crossover of* comparison groups occurs. That is, hospitals initially randomized to

the intervention group will now receive no unsolicited telephone consults when a patient with SAB is included. These patients will also be recorded and followed up. The hospitals that were initially randomized to the control group will then receive a non-solicited telephone consult when a patient with SAB is reported.

4.4 Scope of the study

The study will be conducted multicenter at at least 15 centers in Thuringia. A total of 500 patients will be included. The detailed case number calculation is presented in section 9.2.

4.5 Patient recruitment

As calculated in the "Case Number Planning" section, 550 patients with SAB will be screened and 500 patients will be enrolled in this study. The plan is to recruit this number of participants in at least 15 of the up to 39 centers participating in AlertsNet 2.0. The target number of participants per center is 17 per arm and phase, so that at least 15 patients per arm and phase will be available for evaluation.

With an estimated incidence rate between 22 and 32/100,000 person-years (3), between 500 and 700 SAB cases per year can be expected in Thuringia with a population of 2.2 million inhabitants. The case rate planning is presented in detail in **section 9.2.**

4.6 Schedule

Table 1: Schedule of the SUPPORT study with indication of the individual work packages and milestones

Work package no.	Milestone (▼)	**Year 1** 08/15 – 07/16	**Year 2** 08/16 - 07/17	**Year 3** 08/17 – 07/18
1	Study preparations (preparation of study documents, recruitment and randomization of hospitals) completed.	▼		
2	First patient enrolled	▼		
	50% of the total 500 patients included		▼	
	Follow-up completed, database closed			▼
3	Statistical analysis completed			▼
	Final report and scientific manuscript prepared			▼

Table 2: Description of the individual milestones of the SUPPORT study.

No.	Milestone	Time after project start (months)
▼1	Preparation and establishment of the study.	6
▼2	Inclusion of 1st patient.	10
▼3	Inclusion of the 250th patient.	18
▼4	Inclusion of the last patient.	30
▼5	End of follow-up and closure of the database.	33
▼6	Submission of the scientific manuscript.	36

The SUPPORT study timeline is shown in **Table 1** and the individual milestones are shown in **Table 2.** The expected total study duration is 36 months. No interim evaluation is planned. Enrollment in the study will end with the 500th enrolled patient. The study duration per patient is 90 days. The study ends 90 days after inclusion of the 500th patient. The study is divided into different work packages:

Work package 1: Study preparation

After preparation of the study documents (study protocol, patient information and informed consent) and obtaining the ethics votes, the study folders (TMF, ISF), the Case Report Form (CRF), the study database as well as the recruitment and randomization of the participating hospitals are prepared. In addition, the IT infrastructure for data transfer is set up.

Work package 2: Study implementation

In this project phase, patient inclusion in the participating clinics, telephone consultation with the treating physicians in the clinics, as well as documentation of the data in the CRF and data checking (query management) and follow-up surveys (calls to the patients/primary care physicians) will take place. The study completion of the last patient is planned in month 30.

Work package 3: Study completion

The statistical evaluation and interpretation of the data, the preparation of the final report, the publication of the results in professional journals and their presentation at (scientific) professional events are carried out.

5 Participating study centers

This study will include at least 15 of the up to 39 hospitals participating in AlertsNet 2.0 and a total number of 500 patients. Participating hospitals will be randomized into 2 groups with 2 sequences: A-B or B-A. Here, A represents the intervention and B the control group ("standard of care"). During the intervention, treating physicians at participating hospitals will receive unsolicited telephone consults recommending an evidence-based set of interventions to treat SAB (11).

The condition for participation here is that the laboratory to which a hospital sends its microbiological samples is connected to AlertsNet2.0. With regard to the IT concept, reference is made to the AlertsNet2.0 application.

Qualification of the participating centers or treating physicians beyond routine clinical work is not necessary. The telephone consultation is carried out by a clinical infectiologist or a physician with sufficient infectiological experience. There is no minimum number of patients to be included. The change of the groups takes place individually for each center after inclusion of the first 17 patients or after 12 months at the latest.

5.1 Test center directory

Participating study sites are identified in **Section 14.1.**

5.2 Conditions for participation in the study

A condition for a hospital's participation in the study is the hospital's connection to the AlertsNet platform, which allows the information about the presence of an *S. aureus-positive* blood culture to be sent directly to the SUPPORT study center. There are no restrictions regarding the size of the hospital or the patients treated. The responsibilities of the study centers are specified in cooperation agreements.

Patients participating in the study must meet the inclusion criteria stated in **Section 6.1.** No exclusion criterion must be present (see **Section 6.2**).

6 Patient selection

6.1 Inclusion criteria

All adult (at least 18 years old) patients, regardless of gender, with diagnosed SAB (positive blood culture) who have given their written consent to participate in the study should be included. Re-enrollment of a patient who had already been included in the study will only occur if the 2nd SAB episode occurs more than 90 days after the 1st SAB episode and there is no evidence of recurrence due to deep-seated infection. In this case, the patient will be re-educated and must again consent to study participation. In principle, he will be managed as a new patient. It must be noted in the patient file and in the CRF that this is a 2nd SAB episode.

6.2 Exclusion criteria

Patients will not be included in the study if any of the following criteria are present:

- Positive blood cultures with only other pathogens in the blood culture (patients in whom more than one bacterial species is detected are included if one pathogen is *S. aureus*).
- *S. aureus infections* without bacteremia (e.g., superficial wound infections with evidence of *S. aureus*).
- Patients receiving palliative care, with treatment limitations or cessation (e.g., DNR order), or with life expectancy < 90 days due to underlying disease.

7 Course of studies

7.1 Detailed study process

Information about a microbiological blood culture result *positive* for *S. aureus* is automatically reported to the SUPPORT team via the AlertsNet infrastructure (Electronic Blood Culture Registry, EBCR). However, data management is done separately. The system sends a fax to the treating hospital with the patient's clinical name, -year of birth, *subject identification code* (SIC) and microbiological findings. The study center at the UKJ, which is informed at the same time, does not receive any clear name information but only the information of a SAB detection in a specific institution and the automatically generated case code (SIC). The study center contacts the study physician at the clinical institution and, based on the relevant positive microbiological findings and using the SIC, asks for the patient to be included in the study. If the patient agrees to participate in the study after being informed by the study physician, the signed consent form is faxed to the study center. Only with this fax does the study center thus receive clear name information on the SAB-positive patient. Thereupon, for patients of the intervention arm, the telephone consultation with the treating physician is performed and subsequently the consultation is confirmed by fax (incl. therapy proposal). For patients in the control group (no infectious disease consultation), the two aforementioned steps are omitted. After inclusion in the study, only the informed consent form is faxed to the study center. A consultation is not performed for these patients.

The SUPPORT study nurse documents the data of the study patients pseudonymously in the CRF as soon as possible during her on-site visits. After 30 and after 90 days, a follow-up call is made to the patients by the study center. The contact details for this are recorded separately in the consent form (***Figure*** and **Section 14.2**).

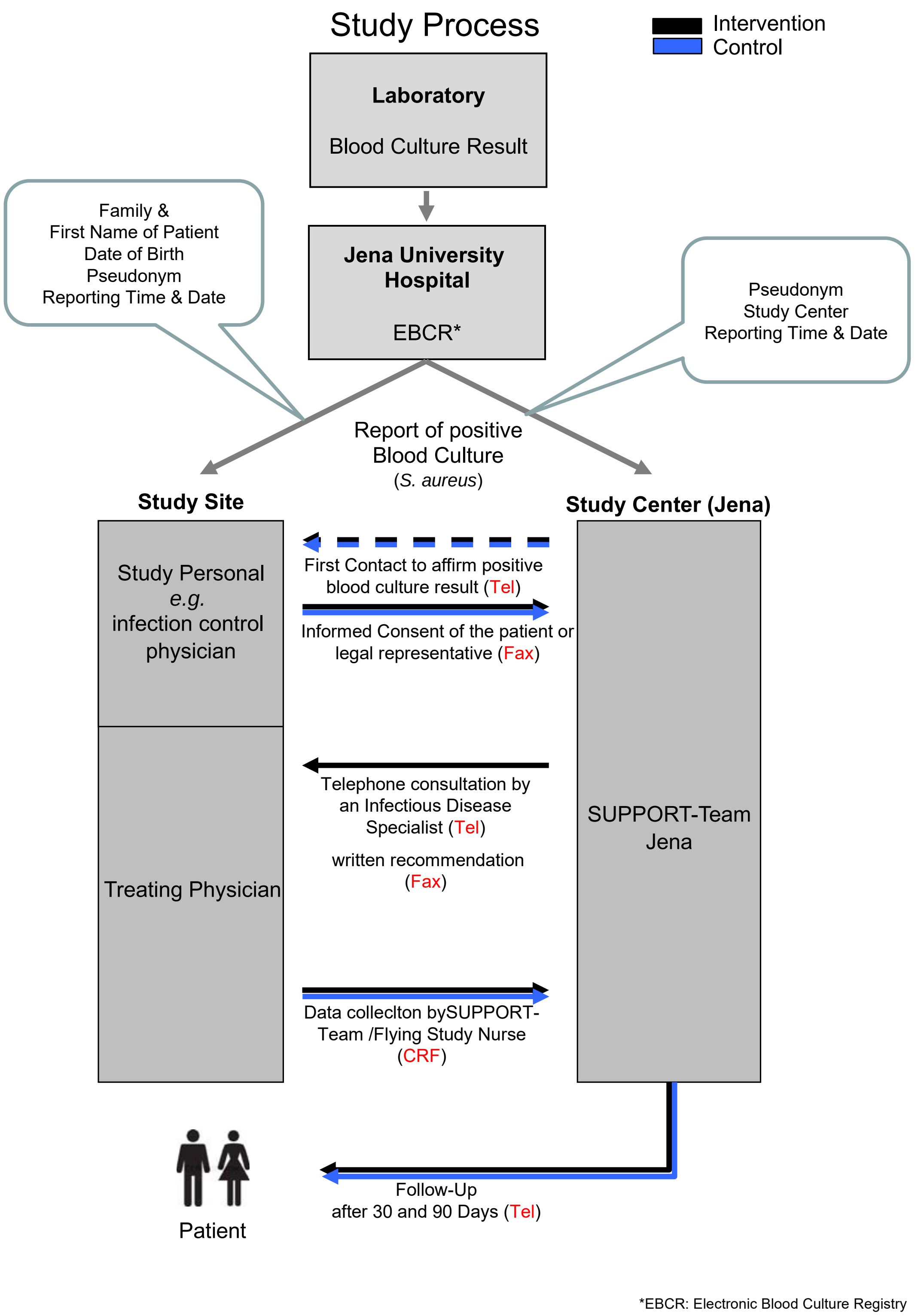

Figure: *Flow chart* for the SUPPORT study.

Checking the inclusion and exclusion criteria

The examination of the inclusion and exclusion criteria of potential study patients ("screening") is performed using a predefined checklist. All patients who meet the inclusion criteria are documented in a central "Screening Log" (**Appendix 2**) at the study center. This log will also indicate whether the patient was included in the study or for what reason, if any, the patient was not included. If a patient fulfills all inclusion criteria and there are no exclusion criteria, he/she is to be entered in the patient identification list with the date of study inclusion.

7.2 Patient education and consent

Each patient is informed about the nature, significance, objectives, possible risks, expected benefits, implications and other aspects of the study by means of a discussion between the study physician and the patient. The patient receives the written patient information (**see Appendix 3**). The study physician makes sure that the patient has understood the information. After the information session, each patient is given sufficient time and opportunity to clarify any unanswered questions and to decide on his or her participation.

Each patient signs and dates his or her consent to participate in the study in writing on the consent form. The patient's consent must also explicitly refer to the collection and processing of personal data. Therefore, patients are explicitly informed about the purpose and scope of the collection and the use of these data, especially health data.

If a patient is unable to sign the consent personally, a witness must be present during the informed consent process who can confirm that the patient has given verbal information and consent by signing.

Since no invasive procedures are performed as part of the study and the study-related intervention is merely counseling and documentation, with the decision to implement the recommendation remaining at the discretion of the treating physician, consent may be obtained from the health care proxy or a court-appointed guardian in the case of a patient who lacks capacity to consent.

One copy of the signed consent form (copy or 2nd original) is given to the patient or caregiver. The other remains at the study center. The SUPPORT study center receives a copy by fax message.

The patient or caregiver may withdraw consent to study participation at any time and without giving reasons. It is requested to state the reason for withdrawal, but it is pointed out that this is a voluntary statement. The time of withdrawal of consent to treatment or study is to be documented in the patient's file by the study team at the center.

The presence of pregnancy is not an exclusion criterion.

If it is subsequently determined that there was a violation of the inclusion and exclusion criteria, the study management decides whether the patient should be included in the statistical evaluation of the "per protocol analysis". The documentation of the patient is independent of this. All included patients are to be included in the ITT analysis.

7.3 Randomization

Randomization of the hospitals to the sequence A-B or B-A will be performed before the start of the study as block randomization with variable block length. It is performed with the help of randomization software at Jena University Hospital by a study-independent person of the

Center for Clinical Studies (ZKS) Jena. The randomization list will be kept inaccessible until the end of the study.

7.4 Treatment phase

A treatment phase in the actual sense does not exist in the SUPPORT study. The patients in the intervention group should be treated according to the infectious diseases consil. The implementation of the consil is the responsibility of the treating physicians of the study centers. Treatment measures carried out are to be documented in the patient file in such a way that the data can be recorded by the SUPPORT study nurse during the on-site visits to the study centers.

7.5 Description of laboratory and other investigations and methods

All medications (beta-lactam antibiotics, vancomycin, etc.) or examinations (e.g., transesophageal echocardiography) recommended in the infectious disease consult are routinely used in clinical practice.

7.6 Examinations within the framework of discharge

No special examinations are scheduled as part of the discharge process.

7.7 Follow-up surveys

The survival of the patients and the recurrence rate will be recorded up to 90 days after diagnosis of SAB. For this purpose, the patients or, if they are not available, the primary care physician or the study centers will be interviewed by telephone 30 and 90 days after diagnosis. Patient visits are not planned.

7.8 Description of the individual visits

No patient visits will be conducted as part of the study.

7.9 End of study participation

The regular study end date for each study participant is 90 days after diagnosis of SAB.

7.10 Premature withdrawal of a patient from the study (discontinuation criteria).

Each participant has the right to withdraw from the study prematurely at any time at his own request and without giving reasons (consent is withdrawn), without any disadvantages for his further (medical) treatment.

If a patient withdraws consent or wishes to discontinue treatment, this must be documented accordingly as a reason for discontinuation, stating the time, in the patient record and in the CRF.

If a patient cannot be contacted at the 30-day follow-up, this does not automatically mean that the study has been discontinued. The 90-day follow-up will still be performed.

7.11 End of the study

7.11.1 Regular end of study

The regular end of the study is defined as the date on which the last patient has completed the follow-up ("Last Patient Out - LPO").

7.11.2 Premature end of study/discontinuation of the entire study

Reasons for premature termination of the entire study may include:

- Decision of the study management in case of unacceptable risks under benefit-risk consideration
- new scientific findings during the term of the study
- non-adequate recruitment rate
- No study inclusion within the first 3 months after the start of recruitment.

The study director informs the lead ethics committee of the reasons for a planned discontinuation of the entire study. This committee decides after detailed consultation; if it approves the application, the study director is obliged to inform the participating study centers immediately.

8 Adverse events/complications

8.1 Adverse events

SUPPORT is not a study in the sense of the AMG. All recommended measures and medications are routinely used in everyday clinical practice. Specific adverse events due to the intervention (telephone consultation) are not expected, since the telephone consultation only suggests treatment options that the treating physician can implement. Complications of the recommended and implemented diagnostic and therapeutic measures (e.g. esophageal perforation after transesophageal echocardiography, contrast medium- or vancomycin-induced renal insufficiency, pneumothorax when changing a CVC, etc.) are documented.) are documented.

8.2 Possible complications and/or risks

Complications are defined as all events that are or could be related to the therapeutic and diagnostic measures recommended in the infectious disease consultation. These include complications that may occur as a result of the medications (intolerances to the recommended antibiotic or contrast agent when performing contrast computed tomography) or as a result of medically interventional complications, such as those that may occur during abscess cleavage or transesophageal echocardiography. The drugs to be recommended are approved for the treatment of *S. aureus* bacteremia by the "European Medicines Agency" (EMA). The medical measures to be recommended belong to routine clinical examinations, about the risks of which the treating physician is obliged to inform the patient before the examination. Therefore, for the SUPPORT study, there are no specific risks for the participating patients that exceed the clinical routine.

8.3 Recording and documentation of complications

Complications are recorded over a period of 90 days after inclusion in the study. In case of doubt, the study outpatient clinic should be contacted by telephone. In case of death, the

cause of death should be documented. Unexpected, previously unknown complications for which a causal relationship with the recommended measures cannot be ruled out will be reported to the study participants within one week.

All complications will be documented in the patient's chart and later recorded in the CRF by the SUPPORT study nurse.

9 Biometrics

9.1 Endpoints

The primary endpoint of the study is 30-day all-cause mortality of patients starting from the time of SAB diagnosis.

The secondary endpoints are:

- Fulfillment of 6 of the defined quality indicators (yes/no).
- 90-day all-cause mortality starting from the time of SAB diagnosis.
- 90-day recurrence rate starting from the time of SAB diagnosis.
- Progression of SAB to sepsis/septic shock within the 90-day period (based on time of SAB diagnosis) (yes/no).
- Presence/development of septic metastases (abscesses, spondylodiscitis, osteomyelitis, endocarditis, etc.) within the 90 days (based on the date of SAB diagnosis) (yes/no).

Other parameters recorded:

Patient demographic parameters, Charlson comorbidity index (*23*). Infection focus, SIRS severity on admission/diagnosis, sensitivity to methicillin, antibiotic therapy performed, Pitt bacteremia score (*24*), cause of infection, primary site of *S. aureus* infection.

9.2 Definition of evaluation collectives

The primary analysis will be performed according to the intention-to-treat (ITT) principle - i.e. in all included patients. Sensitivity analyses will be performed in the per-protocol (PP) collective, for which rules will be defined prior to study completion.

9.3 Planning the scope of the study (case number planning)

Case number planning was performed for the primary end point, 30-day mortality, using the simulation software of Reich et al (*25*). A systematic review and meta-analysis were performed for case number planning (*26*). The meta-analysis, although not including any randomized trials, allowed estimation of the probability of patient death (within the 30 days starting from the time of SAB diagnosis) in the intervention vs. control group. This probability was estimated to be between 0.25 and 0.5 for the intervention group and between 0.5 and 0.7 for the control group. These probabilities translate into a relative risk of ~0.5. To detect such an effect with a power of >80% at a two-sided significance level of α=5% and low to moderate variation between study centers in the given design, at least 15 centers need to include an average of 2x15 patients (30 patients per cluster results in a total case number of 15x30=450 patients to be included). Since the follow-up visits consist only of telephone visits, which can also be answered by relatives of the patients, a low drop-out rate can be expected during the course. In order to be prepared for a possible drop-out, a total number of 500 patients is targeted.

9.4 Interim evaluation

Interim evaluations of the data are not provided.

9.5 Statistical analysis

Differences in the primary end point, 30-day all-cause mortality, are determined confirmatorily within the framework of a generalized linear mixed model (GLMM). This was specified in more detail by Reich et al (25) and also forms the basis of the case number calculation. This model is suitable to represent the dependence structure by the clusters and the period effects for a dichotomous outcome variable, where the null hypothesis of no treatment effect can be rejected if the two-sided p-value of the Wald test statistic for the fixed effect *group* can be rejected at the two-sided significance level α=5%. The confirmatory test is performed according to the ITT principle, with missing endpoint data in included patients replaced by multiple imputation. Further sensitivity analyses of the primary endpoint are "worst case" imputations as well as analyses in the PP collective. In addition, the *a priori* defined subgroups center, gender and age groups (quartiles) of the patients will be analyzed stratified. For these analyses as well as for the adequate statistical analyses of the secondary endpoints, which will also be performed within GLMM, no multiplicity adjustment is planned.

9.6 Presentation of the results

It is planned to publish the data in a peer-reviewed scientific journal after completion of the data analysis. An interim analysis or a publication of data before completion of the study, also in the context of a congress presentation, is not planned. The presentation of data will follow the guidelines of the CONSORT statement (http://www.consort-statement.org) (*27*).

In all publications or presentations of the project, it must be explicitly stated that the financial support is provided by the Federal Ministry of Health (BMG), grant number 01EO1502.

Regarding the rights and obligations of the authors involved, the publication guidelines for authors in medical journals according to the recommendations of the American Medical Association (http://jama.jamanetwork.com/public/instructionsforauthors.aspx) must be followed.

10 Data Management

10.1 Patient Identification List

Each study center maintains a patient identification list in which patient identification numbers (automatically generated SIC) are linked to participants' full patient names, date of birth, gender, and date of study enrollment. This list is used to enable subsequent identification of participating patients.

The patient identification list must be treated confidentially and must not leave the study center. It must be archived for **at least ten years** after the end of the study. In addition, the participation of the persons concerned in the study is noted in the respective patient file.

10.2 List of responsibilities

For each study center, a signature list including the name, function in the study, study-related activity and abbreviation of the responsible persons is filed in the study center folder and in the central trial folder. With this overview, other persons involved in the clinical trial are also designated with their names, signature and abbreviations as well as their responsibilities and authorities.

10.3 Data collection/documentation forms

10.3.1 Data collection

To achieve the study objective, it is necessary to collect and process medical data of the study participants. Data collection is performed by the SUPPORT study nurse at the study centers participating in the study, based on their documentation systems.

Source data in the sense of ICH guideline E6 are defined as all routinely collected data and laboratory reports as well as special CRFs for the documentation of the study. The majority of the data to be collected is routinely collected and entered directly into the regular patient record.

10.3.2 Documentation forms

A documentation sheet for the study is provided to the study centers. The actual documentation of the data will be done by the SUPPORT study nurse at the study centers directly from the medical record. The following data should be entered into the CRF:

10.3.2.1 Study inclusion -Patient identification number

-Inclusion criteria according to **section 6.1**
-Exclusion criteria according to **section 6.2**
-Presence of the declaration of consent
-Patient inclusion (no/yes), date and time if applicable.
-Group Allocation

10.3.2.2 Demographic data

-Age
-Sex
-Height
-Weight
-Date of hospital admission
-Reason for recording
-Stay in other inpatient facilities

10.3.2.3 Resistance of the *S. aureus isolate of* the 1st blood culture.

10.3.2.4 Origin of infection

-ambulatory
-health-care associated
-nosocomial

10.3.2.5 Infection focus

-Intrathoracic infection
-Urogenital/renal infections
-Infection of the central nervous system
-Bone and joint infections
-Infections of the cardiovascular system

-HNO/oropharyngeal infections
-Intraabdominal infections
-Skin and soft tissue infections
-Postoperative wound infections
-Flexure tube as suspected source of infection

10.3.2.6 Characteristics of blood culture

-Date of blood culture collection
-Location of blood culture collection
-Presence of foreign bodies or prostheses
-Further positive blood cultures
-Detection of other bacteria

10.3.2.7 Presence of SIRS criteria

10.3.2.8 Organ dysfunction within 7 days of 1st positive blood culture.

-Acute encephalopathy
-Thrombocytopenia
-Arterial hypoxemia
-Renal dysfunction
-Metabolic acidosis

10.3.2.9 Septic shock within 7 days after 1.positive blood culture.

10.3.2.10 Severity of SAB

-uncomplicated
-complicated

10.3.2.11 PITT score on the day of blood culture collection (*24*).

10.3.2.12 Charlson comobridity index (*23*)

10.3.2.13 Recommended measures/implementation of the recommended measures

-Time
-Follow-up blood cultures
-Focus remediation
-Transesophageal echocardiography
-other proposed measures

10.3.2.14 Laboratory values on the day of the positive blood culture

- Creatinine
-Creatinine Clearance
-Bilirubin
-Lactate
-CRP
-PCT
-ALAT
-ASAT
-AP
-leukocytes
-platelets
-neutrophil granulocytes
-Ferritin

10.3.2.15 Patient status at hospital discharge

-ICD 10 Main diagnosis
-Place of discharge
-Mortality date
-Discharge date
-ITS stay during hospitalization.
-Complications
-Progression of the SAB

10.3.2.16 Complications

-Contrast agent allergy when performing a CT
-Esophageal rupture during transesophageal echocardiography.
-Pneumothorax during catheter reimplantation
-Death during surgical focal rehabilitation.
-Acute renal failure under vancomycin.
-Acute renal failure under aminoglycosides.
-Acute liver failure during flucloxacillin therapy.
-Acute liver failure during rifampicin therapy.
-Antibiotic Allergy
- occurrence of *Clostridium difficile enteritis.*
-other complications

10.3.2.17 30-days follow up

-successful contact
-Recent hospitalization and, if applicable, reason for admission
-Death of the patient
-New-onset clinical complaints.

10.3.2.18 90 days follow up

-Successful contact
-Recent hospitalization and, if applicable, reason for admission
-Death of the patient
-new clinical complaints

10.3.2.19 Antimicrobial therapy since 1st positive blood culture until discharge/transfer.

- • Drug
-Application type
-Application duration

10.4 Data acquisition and processing

Data acquisition is performed either directly or retrospectively (after initial recording of data in a paper CRF) via web application on the servers of the ZKS of the University Hospital Jena into the study management software "OpenClinica®". The software complies with regulatory requirements (GCP, 21CFR Part11). The data are retrospectively collected via an encrypted data connection (HTTPS) in input masks via web browser or, if this is not possible primarily via a paper CRF by a trained study nurse of the Site Management Unit of the CSCC, in the study centers. To ensure pseudonymous data analysis, a unique patient identification number (SIC) is assigned to each patient.

Data management is performed using the same software. Verification of the accuracy of the data is performed by range, validity and consistency checks during data entry. Non-plausible or missing data are clarified by the SUPPORT study nurse on site with the responsible staff in the study centers. Every change to the data, e.g. due to the incorporation of answered

queries at the centers, is documented in the database via an automatic change tracking (audit trail). By using a hierarchical access concept based on user roles, unauthorized access to the study data is not possible.

Backups of electronic data on the University Hospital servers are performed on a regular basis. The data storage (servers) are located in a separate locked room to which only designated system administrators have access. The database containing patient contact information is secured for access in the same way as the database containing clinical patient data; storage is on two different servers for data protection reasons.

The microbiological findings data are recorded within the framework of the AlertsNet infrastructure (Electronic Blood Culture Registry, EBCR). This process is described in more detail below.

The microbiology laboratory that examines the blood cultures sent in creates a laboratory report for each blood culture and sends it back to the clinic concerned. The findings are also recorded in the AlertsNet EBCR. After this, a semi-automated data exchange begins, which in the case of clinically relevant positive findings provides for the request for documentation of clinical data on the patient concerned by fax to the clinical facility (see AlertsNet technical IT concept version 4.3). If *S. aureus* is detected, in addition to informing the sending hospital, a fax message is automatically generated for the SUPPORT study center. For data protection reasons, this fax only contains pseudonymized data - personal data are only transmitted from the study centers to the SUPPORT study center after the patient has given his/her consent. A patient pseudonym is automatically generated for each finding/database entry (SIC; see **Section 10.1**). In addition to the SIC and the time of reporting, the clinical study center receives the clear names by fax; the SUPPORT study center in Jena receives only the SIC by fax. Only when the SAB-positive patient has consented to study participation does the SUPPORT study center receive his or her name and contact details for the planned telephone follow-up interview with a fax copy of the consent form (see study procedure in Section 7.1).

The transmission of personal data takes place exclusively via https, VPN connections or by fax.

10.5 Study documents and their storage (archiving)

The relevant study documents according to ICH-GCP are compiled and maintained in the central study folder (Trial Master File / TMF) in the study center and kept during the course of the study. The TMF as well as the Investigator Site File (ISF) are prepared by the SUPPORT study nurse together with the project management based on the applicable Standard Operating Procedures (SOPs) of the University Hospital Jena.

The investigator folders are handed over to the staff responsible for the study at the study centers during the initial visits. The study centers are responsible for maintaining them during the course of the study and for archiving them. The SUPPORT study nurse at the study center will check them at regular intervals and especially before study completion to ensure that they are up to date and complete. She will also inform the study teams before study completion to archive the study documents according to the applicable guidelines.

The archiving of the Central Study Folder (TMF) takes place at Jena University Hospital, taking into account the applicable SOPs. The preparation for its archiving is organized by the project management together with the SUPPORT study nurse. The archiving period is at least ten years after preparation of the final report. Study documents shall be retained in accordance with the archiving period applicable to the study centers, but not less than ten years. The study site or the investigator-in-charge must take precautions that prevent the accidental or premature destruction of these documents.

10.6 Privacy

As part of the study, it is necessary to collect and process personal data from the study participants (e.g., full name, initials of first and last name, date of birth, address) and data on treatment and the course of the disease (e.g., medical findings, types of treatment, prescribed medication). These data are collected at the study centers and stored electronically in pseudonymized form (i.e., without direct reference to the patient's name) using a patient identification number. The data will then be extracted at the centers by the SUPPORT study nurse and entered into the CRF, transmitted to the study center, reviewed and analyzed. In the event of withdrawal of consent to the study by the patient, including further data collection, no further data will be collected from the date of withdrawal. The data collected so far will continue to be used and evaluated within the study. If a patient only discontinues the study treatment, the data required for the study can continue to be collected and used.

11 Quality assurance

11.1 Standardization

The study management prepares a study manual describing the study-specific procedures, time windows and responsibilities ("Working Instructions") for the staff involved in the study implementation. This is made available to the study centers when the study is initiated (initial visit to the center); training on the use of the documents is also provided by the SUPPORT study nurse at the study center.

11.2 Control of the study process and data quality

The SUPPORT study nurse visits the participating study centers at appropriate intervals and randomly checks compliance with the requirements of the study protocol and the applicable guidelines (GCP). In this context, she checks in particular the study folder for up-to-dateness and completeness of the essential study documents and the documentation on patient screening and recruitment.

The SUPPORT study nurse at the study center enters the study data into the electronic database during her regular center visits. She is supported by the study teams in the clinics with regard to access to the source data of the study patients.

11.3 Monitoring data

Independent monitoring by the Center for Clinical Studies of the University Hospital Jena is not planned. Compliance with the study protocol and applicable guidelines (GCP) is regularly and randomly checked by the SUPPORT study nurse of the study center (see **paragraph 11.2**).

11.4 Audits

The central quality management of the Jena University Hospital, the project and quality manager and/or the study coordinator of the CSCC may perform internal audits to check the process, documentation and completion of the study according to the study protocol and applicable guidelines (Declaration of Helsinki, GCP).

12 Ethical concerns, legal and administrative regulations

12.1 Declaration of Helsinki and Good Clinical Practice

The study will be conducted in accordance with the ethical principles originating in the Declaration of Helsinki (*28*). The current version of the Declaration will be observed. The recommendations of Good Clinical Practice (http://www.dgrw-online.de/files/leitlinien_gcp_korrektur_2002_deutsche_version.pdf) valid since 17.1.1997, will be taken into account as far as applicable for SUPPORT.

12.2 Ethics Committees

The study protocol, together with the other required documents, will be submitted to the responsible lead ethics committee (Ethics Committee of Jena University Hospital) with a request for evaluation and subsequently to the Ethics Committee of the Thuringian Medical Association (LÄK). The study can only begin after a positive evaluation without conditions by both ethics committees.

The ISFs of the participating investigational sites will each receive a copy of the approving evaluations of the ethics committees.

12.3 Subsequent changes

The study protocol must be adhered to. Changes or additions to the study protocol can only be initiated and authorized by the study management. The lead ethics committee and the ethics committees of the participating study centers will be informed of any changes to the study protocol. If necessary, the approving evaluation will be obtained again. Changes requiring evaluation may not be implemented before the decision of the ethics committee.

Changes to the study that have been approved by the Ethics Committee that are appropriate,

- have an impact on the safety of the persons concerned,
- Additional data collection or analysis that requires a change in patient information and/or consent,
- Influence the interpretation of the scientific documents on which the study is based or the scientific validity of the study results,
- Substantially change the way the study is managed or conducted,

may only be made if these changes have been approved by the ethics committees.

12.4 Legal regulations

The planned study is not a study according to the German Drug Law, Medical Devices Law, X-Ray Ordinance or Radiation Protection Ordinance.

12.5 Patient Insurance

The conclusion of a separate patient insurance is not necessary, since no study-related invasive measures are performed on the patient.

12.6 Registration

The study is to be registered in the following public study registers: German Clinical Trials Register (*DRKS).* The study director is responsible for the registration in the registry and the maintenance of the registry data.

12.7 Funding

The study is funded by the German Federal Ministry of Education and Research (BMBF), FKZ 01EO1502, as part of the second funding period of the Integrated Research and Treatment Center (IFB) "Sepsis and Sepsis Consequences" (CSCC 2.0) at Jena University Hospital. Further external funding is not planned for the study.

12.8 Final report and publication

In preparing the final report and publication, the standards of the CONSORT Statement (http://www.consort-statement.org), the SQUIRE Statement (http://www.squire-statement.org) will be considered, as well as the guidelines for authors in medical journals as recommended by JAMA (http://jama.jamanetwork.com/public/instructionsforauthors.aspx).

For publications, authors are required to refer to the study as follows:

University Hospital Jena, Integrated Research and Treatment Center "Sepsis and Sepsis Consequences (CSCC 2.0)". This work was funded by the German Federal Ministry of Education and Research (BMBF), FKZ 01EO1502.

respectively

Jena University Hospital, Integrated Research and Treatment Center - Center for Sepsis Control and Care (CSCC 2.0). This work was supported by the Federal Ministry of Education and Research (BMBF), Germany, FKZ 01EO1502.

Abstracts that can be cited must refer to the CSCC and the sponsor in the same way. In addition, the logo of the CSCC and the BMBF must be used on posters. The publication of the study results is independent of the results.

13 Literature

1. W. V. Kern, Management of Staphylococcus aureus bacteremia and endocarditis: progresses and challenges. *Current opinion in infectious diseases* **23**, 346-358 (2010).
2. K. B. Laupland, Incidence of bloodstream infection: a review of population-based studies. *Clinical microbiology and infection : the official publication of the European Society of Clinical Microbiology and Infectious Diseases* **19**, 492-500 (2013).
3. K. B. Laupland *et al* , The changing epidemiology of Staphylococcus aureus bloodstream infection: a multinational population-based surveillance study. *Clinical microbiology and infection : the official publication of the European Society of Clinical Microbiology and Infectious Diseases* **19**, 465-471 (2013).
4. N. Mejer *et al.* , Stable incidence and continued improvement in short term mortality of Staphylococcus aureus bacteraemia between 1995 and 2008. *BMC infectious diseases* **12**, 260 (2012).
5. D. J. Hetem *et al* , Preventing Staphylococcus aureus bacteremia and sepsis in patients with Staphylococcus aureus colonization of intravascular catheters: a retrospective multicenter study and meta-analysis. *Medicine* **90**, 284-288 (2011).
6. C. Big, P. N. Malani, Staphylococcus aureus bloodstream infections in older adults: clinical outcomes and risk factors for in-hospital mortality. *Journal of the American Geriatrics Society* **58**, 300-305 (2010).
7. D. H. Mitchell, B. P. Howden, Diagnosis and management of Staphylococcus aureus bacteraemia. *Internal medicine journal* **35 Suppl 2**, S17-24 (2005).
8. S. Schmitt *et al.* , Infectious diseases specialty intervention is associated with decreased mortality and lower healthcare costs. *Clinical infectious diseases : an official publication of the Infectious Diseases Society of America* **58**, 22-28 (2014).
9. H. Honda, M. J. Krauss, J. C. Jones, M. A. Olsen, D. K. Warren, The value of infectious diseases consultation in Staphylococcus aureus bacteremia. *The American journal of medicine* **123**, 631-637 (2010).
10. J. O. Robinson *et al.* , Formal infectious diseases consultation is associated with decreased mortality in Staphylococcus aureus bacteraemia. *European journal of clinical microbiology & infectious diseases : official publication of the European Society of Clinical Microbiology 31,* **2421-2428** (2012).
11. L. E. Lopez-Cortes *et al* , Impact of an evidence-based bundle intervention in the quality-of-care management and outcome of Staphylococcus aureus bacteremia. *Clinical infectious diseases : an official publication of the Infectious Diseases Society of America* **57**, 1225-1233 (2013).
12. R. V. Rasmussen *et al.* , Prevalence of infective endocarditis in patients with Staphylococcus aureus bacteraemia: the value of screening with echocardiography. *European journal of echocardiography : the journal of the Working Group on Echocardiography of the European Society of Cardiology* **12**, 414-420 (2011).
13. A. G. Jensen, Importance of focus identification in the treatment of Staphylococcus aureus bacteraemia. *The Journal of hospital infection* **52**, 29-36 (2002).
14. T. Lahey, R. Shah, J. Gittzus, J. Schwartzman, K. Kirkland, Infectious diseases consultation lowers mortality from Staphylococcus aureus bacteremia. *Medicine* **88**, 263-267 (2009).
15. M. Nagao *et al.* , Close cooperation between infectious disease physicians and attending physicians can result in better management and outcome for patients with Staphylococcus aureus bacteraemia. *Clinical microbiology and infection : the official publication of the European Society of Clinical Microbiology and Infectious Diseases* **16**, 1783-1788 (2010).

16. A. A. Pragman, M. A. Kuskowski, J. M. Abraham, G. A. Filice, Infectious disease consultation for Staphylococcus aureus bacteremia improves patient management and outcomes. *Infectious diseases in clinical practice* **20**, 261-267 (2012).
17. S. Rieg *et al.* , Mortality of S. aureus bacteremia and infectious diseases specialist consultation--a study of 521 patients in Germany. *The Journal of infection* **59**, 232-239 (2009).
18. S. Weis *et al* , [Staphylococcus aureus bacteremia - a distinct entity]. *Deutsche medizinische Wochenschrift* **140**, 982-989 (2015).
19. A. G. Jensen *et al* , Treatment and outcome of Staphylococcus aureus bacteremia: a prospective study of 278 cases. *Archives of internal medicine* **162**, 25-32 (2002).
20. A. J. Kaasch *et al* , Staphylococcus aureus bloodstream infection: a pooled analysis of five prospective, observational studies. *The Journal of infection* **68**, 242-251 (2014).
21. S. H. Kim *et al*, , Outcome of vancomycin treatment in patients with methicillin-susceptible Staphylococcus aureus bacteremia. *Antimicrobial agents and chemotherapy* **52**, 192-197 (2008).
22. M. Vogel *et al.* , Impact of infectious diseases specialists consultation on the management and outcomes of Staphylococcus aureus bacteremia - a systematic review and metaanalysis. *Poster Abstract ECCMID Annual Meeting*, (2015).
23. M. E. Charlson, P. Pompei, K. L. Ales, C. R. MacKenzie, A new method of classifying prognostic comorbidity in longitudinal studies: development and validation. *Journal of chronic diseases* **40**, 373-383 (1987).
24. J. Y. Rhee *et al*, Scoring systems for prediction of mortality in patients with intensive care unit-acquired sepsis: a comparison of the Pitt bacteremia score and the Acute Physiology and Chronic Health Evaluation II scoring systems. *Shock* **31**, 146-150 (2009).
25. N. G. Reich, J. A. Myers, D. Obeng, A. M. Milstone, T. M. Perl, Empirical power and sample size calculations for cluster-randomized and cluster-randomized crossover studies. *PLoS One* **7**, e35564 (2012).
26. M. Vogel *et al.* , Infectious disease consultation for Staphylococcus aureus bacteremia - A systematic review and meta-analysis. *The Journal of infection*, (2015).
27. K. F. Schulz, D. G. Altman, D. Moher, C. Group, CONSORT 2010 Statement: updated guidelines for reporting parallel group randomised trials. *Trials* **11**, 32 (2010).
28. WMA General Assembly, WMA Declaration of Helsinki - Ethical Principles for Medical Research Involving Human Subjects. (2013).

14 Attachments

14.1 List of participating hospitals

The following clinical and rehabilitation facilities participate in AlertsNet2.0.

Clinical facilities-already connected

Robert Koch Hospital ApoldaApolda
HELIOS Clinics Blankenhain / Bleicherode / Gotha / Erfurt / Meiningen
St. Georg-Klinikum EisenachEisenach
Kath. Hospital St. Johann NepomukErfurt
Henneberg ClinicsHildburghausen
University Hospital JenaJena
Elisabeth Hospital SchmalkaldenSchmalkalden
SRH Zentralklinikum SuhlSuhl

Clinical facilities connection in preparation

Altenburger Land ClinicAltenburg
Ilm District Clinics Arnstadt / Ilmenau
Marienstift ArnstadtArnstadt
Zentralklinik Bad Berka (Rhön) Bad Berka
DRK-Manniske HospitalsBad Frankenhausen / Sömmerda / Sondershausen
Hufeland ClinicsBad Langensalza / Mühlhausen
Clinical Center Bad SalzungenBad Salzungen
HELIOS Clinics Hildburghausen
SRH Clinic GeraGera
Greiz District HospitalGreiz
Eichsfeld ClinicKleinbartloff
Ecumenical Hainich ClinicMühlhausen
Südharz-KrankenhausNordhausen
Thuringia Clinics "Georgius Agricola " Saalfeld/Rudolstadt/Pößneck / Neuhaus
MEDINOS Clinics of the Sonneberg DistrictSonneberg
Sophien- und Hufeland-KlinikumWeimar

Clinical facilities contract negotiations ongoing

SRH Waldklinikum EisenbergEisenberg
Schleiz District HospitalSchleiz

14.2 Data protection aspects

Data protection aspects of the flow chart of the SUPPORT study (see Technical IT Concept Version 4.3 AlertsNet, vote of the Thuringian Commissioner for Data Protection and Freedom of Information (TLfDI) dated April 10, 2015 (AZ: 278-7/2014.42)).

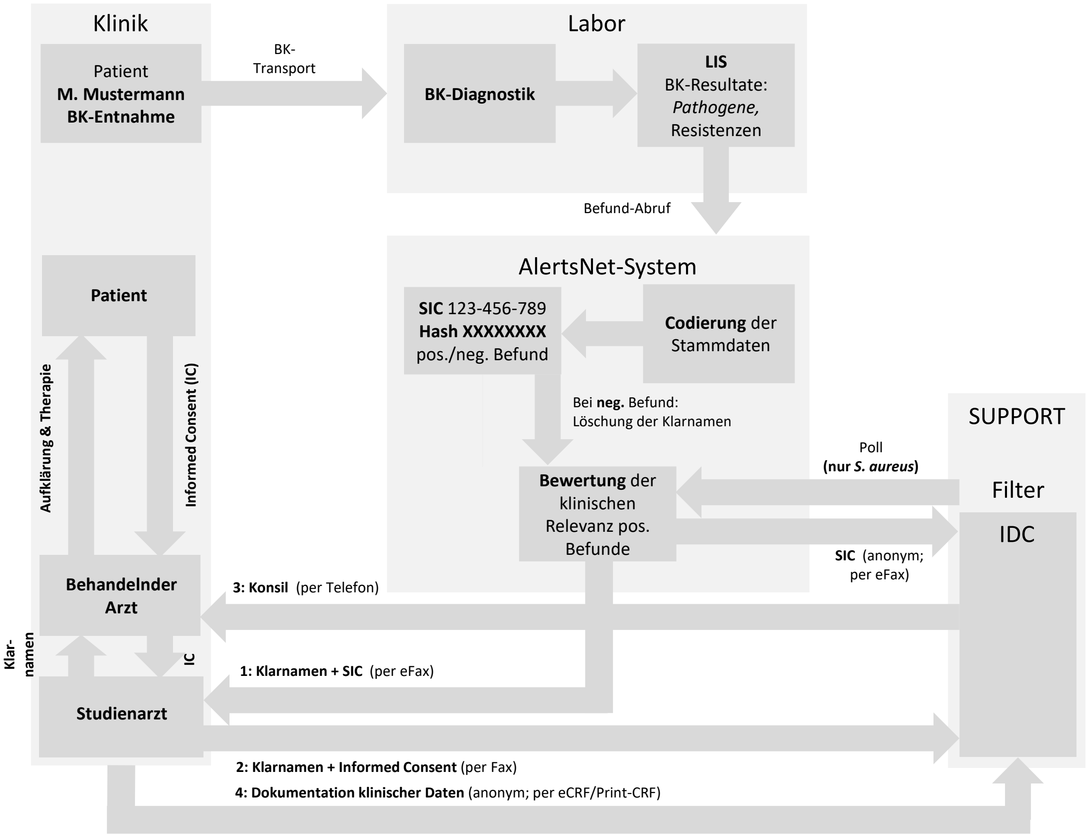

Addendum #1 to the study protocol

SUPPORT

Study of the utility of a statewide counseling program to improve outcomes in patients with *Staphylococcus aureus bacteremia* (SUPPORT study) -.

A single-blind, cluster-randomized trial in a cross-over design.

Study acronym:	SUPPORT
Addendum to the protocol:	Version 1.07 from 18.05.2016

Vote of the Ethics Committee of the University Hospital Jena
Ref. 4608-11/15 from 24.05.2016

Statement of the Ethics Committee of the LÄK Thüringen
Ref. 53394/2016/72 of 16.06.2016

Confidentiality Notice: The contents of this Addendum are to be kept strictly confidential and may not be disclosed to uninvolved parties, either orally or in writing, without the consent of the study director.

1.1 Additions to the recording of a SAB case

In addition to the procedure for automated reporting of a *Staphylococcus aureus-positive* microbiological blood culture finding via the AlertsNet infrastructure (Electronic Blood Culture Registry, EBCR) to the SUPPORT team as specified in study protocol version 1.07 dated May 18, 2016, the report can also be made by telephone or fax by a member of the microbiology laboratory staff. If necessary, this allows for faster processing of the SAB findings message or earlier consultation, since findings are only transmitted via the EBCR at 24-hour intervals. In addition, at study sites where connection to AlertsNet is delayed, SUPPORT study enrollment can be initiated without being tied to the EBCR.

As a rule, the message about the presence of a SAB case in a participating clinical institution is transmitted to the SUPPORT team before obtaining informed consent from the attending physician. In this case, no identifying information is to be forwarded, i.e., in a transmission of the microbiology report by fax, this data is to be blacked out. In individual facilities, consent to study participation may have already been obtained by early contact of the laboratory with the informing physician before the SUPPORT team was informed or an automated receipt of findings via the EBCR took place.

Since, in the case of premature transmission of findings, no sequential number (case code, SIC) can be generated automatically for the procedure, or if a connection to the AlertsNet EBCR is not yet available, an SIC is assigned manually, which can subsequently and after import of the findings message into the AlertsNet EBCR be matched with the then automatically generated SIC.

All further steps, beginning with the study center contacting the study physician at the clinical facility to obtain informed consent (if not already obtained), will follow the study protocol.

1.2 Introduction of a wash out phase

In addition to the specifications made in the study protocol (version 1.07 of 18.05.2016) regarding randomization and inclusion of patients in the intervention and control groups, a one-month wash-out phase will be introduced between the end of the intervention phase (after the inclusion of 15 patients in one institution) and the start of the control phase (before the inclusion of 15 additional patients in the institution who are educated but do not receive a consil). This phase is intended to ensure that knowledge of improved treatment strategies gained during the intervention phase approaches pre-study routines, allowing clearer differentiation between outcomes the intervention and control phases. Accordingly, no wash out phase is required between the end of the control phase and a subsequent intervention phase.

Prof. Dr.med. ***Mathias Pletz***, Head of the study   Date

Sebastian Weis, *M.D.*, Deputy Study Director   Date

Prof. Dr.med. ***André Scherag****, Biometrician* Date

Addendum #2 to the study protocol

SUPPORT

Study of the utility of a statewide counseling program to improve outcomes in patients with *Staphylococcus aureus bacteremia* (SUPPORT study) -.

A single-blind, cluster-randomized trial in a cross-over design.

Study acronym:	SUPPORT
Addendum to the protocol:	Version 1.09 from 25.07.2016

Vote of the Ethics Committee of the University Hospital Jena
Ref. 4608-11/15 from 24.05.2016

Statement of the Ethics Committee of the LÄK Thüringen
Ref. 53394/2016/72 of 16.06.2016

Confidentiality Notice:	The contents of this Addendum are to be kept strictly confidential and may not be disclosed to uninvolved parties, either orally or in writing, without the consent of the study director.

1.1 Extension of the Secondary Endpoints

In addition to the procedure of following up patients after 30 and 90 days as defined in the study protocol version 1.09 dated 25/07/2016 under 1.4., 3.2., 4.6, 7.1, 7.6, it is planned to capture the long-term survival of patients by follow-up telephone calls also 18 months after the diagnosis of *S.aureus* bacteremia. The data to be collected correspond to those of the 30- and 90-day follow-up (successful contact, new hospitalization and, if applicable, reason for admission, death of the patient, new clinical complaints).

The patient information was modified accordingly. For the patients included so far, consent is requested on the phone and documented accordingly. If no verbal consent is given, the patient will be censored at 18 months. The evaluation of long-term survival is performed separately and is not part of the primary evaluation.

Prof. Dr.med. ***Mathias Pletz***, Head of the study Date

Priv.Doz. Dr.med. ***Sebastian Weis***, Deputy Head of the Study Date
