# Supplementary material for: Effect of Automated Telephone Infectious Disease Consultations to Nonacademic Hospitals on 30-Day Mortality Among Patients With Staphylococcus aureus Bacteremia: The SUPPORT Cluster Randomized Clinical Trial
Source: JAMA Netw Open. 2022 Jun 24;5(6):e2218515. doi: 10.1001/jamanetworkopen.2022.18515 (PMC9233240; doi:10.1001/jamanetworkopen.2022.18515)
Supplement: Supplement 2. — eMethods. Supplementary Methods eTable 1. Patient Characteristics at the Cluster Level eTable 2. Additional Patient Characteristics at the Patient Level (Not Cluster Adjusted) eTable 3. Additional Patient Characteristics at the Cluster Level eTable 4. Treatment-Related Adverse Events Reported at the Patient Level (Not Cluster Adjusted) eTable 5. Exploratory Analysis of the Quality-of-Care Indicators Depending on the Order of Study eFigure. Exploratory Analysis of 18-Month Survival With Kaplan-Meier Plots for Individual Participants From (A) All Centers, (B) Centers Without Infectious Disease Consultation (IDC), and (C) for the Composite (Survival and No Readmission) for All Centers [file jamanetwopen-e2218515-s002.pdf]

Supplementary Online Content

Weis S, Hagel S, Palm J, et al; SUPPORT Study Group. Effect of automated telephone infectious disease consultations to nonacademic hospitals on 30-day mortality among patients with *Staphylococcus aureus* bacteremia: the SUPPORT cluster randomized clinical trial. *JAMA Netw Open*. 2022;5(6):e2218515. doi:10.1001/jamanetworkopen.2022.18515

eMethods. Supplementary Methods
eTable 1. Patient Characteristics at the Cluster Level
eTable 2. Additional Patient Characteristics at the Patient Level (Not Cluster Adjusted)
eTable 3. Additional Patient Characteristics at the Cluster Level
eTable 4. Treatment-Related Adverse Events Reported at the Patient Level (Not Cluster Adjusted)
eTable 5. Exploratory Analysis of the Quality-of-Care Indicators Depending on the Order of Study
eFigure. Exploratory Analysis of 18-Month Survival With Kaplan-Meier Plots for Individual Participants From (A) All Centers, (B) Centers Without Infectious Disease Consultation (IDC), and (C) for the Composite (Survival and No Readmission) for All Centers

This supplementary material has been provided by the authors to give readers additional information about their work.

© 2022 Weis S et al. *JAMA Network Open.*

eMethods. Supplementary Methods

Patient Follow-up

Recurrence was defined as a positive *S.aureus* blood culture after negative blood cultures. Loss to follow-up in the analyses indicates that the respective endpoint could not be observed, usually because the patient could not be reached after 30, 90 days or 18 months (*e.g.* to assess whether he or she had died). In the primary analysis those patients were excluded, in the explorative Kaplan-Meier analysis they were censored at the time of the last observation

Adverse Events and Complications

Complications were defined as all events that were or could be related to the therapeutic and diagnostic measures recommended in the infectious disease consultation. We specifically assessed *i.*) contrast agent allergy when performing a CT, *ii.*) esophageal rupture during transesophageal echocardiography, *iii.*) pneumothorax during catheter reimplantation, *iv.*) death during surgical focus sanitation, *v.*) acute renal failure in patients with vancomycin therapy, *vi.*) acute renal failure in patients with aminoglycoside therapy, *vii.*) acute liver failure in patients with flucloxacillin therapy, *viii.*) acute liver failure in patients with rifampicin therapy, *ix.*) allergic reaction to any given antibiotic and *x.*) occurrence of *Clostridioides difficile* enteritis. In addition, unexpected, previously unknown complications for which a causal relationship with the recommended measures could not be ruled out were documented. Renal insufficiency was assessed using the Risk, Injury, Failure, Loss of kidney function, and End-stage kidney disease (RIFLE) score [1].Liver failure was assessed using the Drug Induced Liver Injury (DILI) [2].

Statistical Analysis

For certain outcomes, we report the Relative Quality Improvement (RQI).This indicates the difference (improvement) in mean QI-score between control and IDC group, relative to the QI score in the control group, i.e, (mean(QI_idc) – mean(QI_control))/mean(QI_control). It is effectively a standardized mean difference, where positive values indicate an improvement when switching from control to IDC group.

We conducted one confirmatory test for the primary outcome such that the type I error in strong sense is controlled a level α=5% (two-sided). All other analyses - including those related to secondary outcomes - are exploratory (and some of which as *post hoc*); all results of the exploratory analyses were not adjusted for multiplicity.

eReferences

1. Bellomo R, Ronco C, Kellum JA, Mehta RL, Palevsky P, Acute Dialysis Quality Initiative w. Acute renal failure - definition, outcome measures, animal models, fluid therapy and information technology needs: the Second International Consensus Conference of the Acute Dialysis Quality Initiative (ADQI) Group. *Crit Care*. Aug 2004;8(4):R204-12. doi:10.1186/cc2872

2. *LiverTox: Clinical and Research Information on Drug-Induced Liver Injury*. 2012. (accessed online: 19.07.2016).

eTable 1. Patient Characteristics at the Cluster Level

**Characteristics**	**IDC group**, mean [S.D.]	**Control group**, mean [S.D.]
**Age**	70.19 [5.91]	73.55 [3.81]
		**Sex**
Male	67.08 [17.70]	69.15 [15.41]
Female	32.92 [17.70]	30.85 [15.41]
**Antimicrobial Resistance**		
Oxacillin/Methicillin	5.78 [7.72]	3.08 [5.12]
Rifampicin	0 [0]	0 [0]
Fosfomycin	1.15 [2.57]	0 [0]
Daptomycin	0 [0]	0 [0]
Fluoroquinolones	13.99 [10.94]	13.81 [10.84]
Linezolid	0 [0]	0 [0]
**Implants**		
Hip prosthesis	5.21 [5.87]	9.02 [10.21]
Knee prosthesis	3.65 [5.37]	9.95 [11.07]
Cardiac valve prosthesis	0 [0]	0.59 [2.43]
Pacemaker	11.92 [11.76]	12.88 [8.93]
ICD	6.07 [8.49]	3.92 [8.75]
**Catheters**		
Central venous line	9.39 [10.14]	6.9 [6.18]
Shaldon/PICC/tunneled	10.24 [9.89]	5.52 [5.86]
Port	12.64 [24.09]	6.81 [7.33]
**Mode of Acquisition** [1]		
Community-acquired	34.13 [19.37]	40.91 [21.02]
Health-care associated	8.61 [10.04]	10.13 [9.09]
Nosocomial	57.26 [21.51]	48.96 [21.16]
**Infection Focus** [2]		
Intrathoracic	24.68 [23.52]	15.01 [12.28]
Urogenital/renal	17.59 [22.89]	15.28 [11.83]
Central nervous system	0.74 [3.03]	1.21 [2.69]
Bone/joint	15.01 [14.85]	24.08 [15.03]
Cardiovascular	2.48 [4.15]	6.8 [8.92]
Otolaryngology	0.36 [1.50]	0 [0]
Intra-abdominal	2.8 [5.35]	4.62 [6.87]
Skin/soft tissue	38.72 [22.21]	37.75 [20.65]
Postsurgical wound infection	6.17 [6.39]	13.72 [8.49]

© 2022 Weis S et al. *JAMA Network Open.*

Peripheral line suspected as focus	14.34 [16.53]	6.05 [7.09]
Other catheter-related infection	23.03 [26.00]	14.96 [11.03]
**PITT Score**	0.87 [0.41]	0.91 [0.42]
**Charlson Comorbidity Score**	3.08 [1.18]	3.08 [0.99]
**Polymicrobial Infection**	2.66 [4.51]	3.11 [4.58]
**Complicated SAB**	46.6 [27.32]	53.69 [18.94]
Endocarditis/sept. Metastasis	24.46 [17.76]	26.97 [22.76]
Implants	85.85 [17.76]	84.92 [17.89]
Follow-up blood culture data available	0.51 [0.27]	0.35 [0.18]
Positive follow-up blood culture on day 2-4	27.79 [26.37]	43.81 [30.56]
Fever within 72 h after therapy initiation	15.5 [16.84]	23.04 [12.78]
Remaining catheter in patients with catheter-related infection (total missing: 271)	19 [30.12]	30 [30.97]
**Length of Hospital Stay**	24.33 [6.52]	24.48 [6.53]
**Time from First BC to IDC**	6.9 [2.55]	Not applicable

[1] Community-acquired: signs of infection are present that are judged to be pre-hospital or <48h after the start of hospitalization (without criteria for health care system associated SAB). Health care-associated: in hospital <48 h after admission + infusion therapy/wound care/close care by nurse or family member (within 30 days prior to SAB) or outpatient presentation to a hospital/ hemodialysis practice/ receipt of *i.v.* chemotherapy within 30 days prior to SAB or intravenous continuous medication at home or placement in a nursing home or stay in an acute care hospital for at least 1 day within 90 days prior to bloodstream infection. Nosocomial: no evidence (>48 h after admission to the hospital) that the infection was present or was in the incubation phase before admission to the hospital.

[2] Patient could present with more than one (suspected) focus.

© 2022 Weis S et al. *JAMA Network Open.*

eTable 2. Additional Patient Characteristics at the Patient Level (Not Cluster Adjusted)

All numbers are presented as No. (%) unless otherwise stated.

**Characteristics**	**All patients (n=386)**, N (%)	**IDC group (n= 177)**, N (%)	**Control group (n=209)**, N (%)
**Focus**			
**Intrathoracic**	66 (17.1)	39 (22)	27 (12.9)
Bronchitis	10 (2.6)	6 (3.4)	4 (1.9)
Pneumonia	49 (12.7)	27 (15.3)	22(10.5)
Mediastinitis	2 (0.5)	2 (1.1)	0 (0)
Empyema	3 (0.8)	2 (1.1)	1 (0.5)
Abscess	0 (0)	0 (0)	0 (0)
Other	3 (0.8)	3 (1.7)	0 (0)
**Urogenital**	55 (14.2)	24 (13.6)	31 (14.8)
Urinary tract	53 (13.7)	24 (13.6)	29 (13.9)
Reproductive system	1 (0.3)	0 (0)	1 (0.5)
Other	2 (0.5)	0 (0)	2 (1)
**Central Nervous System**	5 (1.3)	2 (1.1)	3 (1.4)
Meningitis	2 (0.5)	1 (0.6)	1 (0.5)
Abscess	4 (1)	2 (1.1)	2 (1)
Other	0 (0)	0 (0)	0 (0)
**Bone/Joint**	85 (22)	36 (20.3)	49 (23.4)
Spondylodiscitis	28 (7.3)	16 (9)	12 (5.7)
Osteomyelitis	27 (7)	12 (6.8)	15 (7.2)
Joint	23 (6)	8 (4.5)	15 (7.2)
Hip	1 (0.3)	1 (0.6)	0 (0)
Prosthetic hip	3 (0.8)	1 (0.6)	2 (1)
Knee	3 (0.8)	2 (1.1)	1 (0.5)
Prosthetic knee	11 (2.8)	2 (1.1)	9 (4.3)
Spine implant	0 (0)	0 (0)	0 (0)
Other	18 (4.7)	6 (3.4)	12 (5.7)
**Cardiovascular System**	20 (5.2)	6 (3.4)	14 (6.7)
Endocarditis	16 (4.1)	3 (1.7)	13 (6.2)
Native Tricuspid Valve	5 (1.3)	1 (0.6)	4 (1.9)
Prosthetic Tricuspid Valve	0 (0)	0 (0)	0 (0)
Native Mitral Valve	7 (1.8)	1 (0.6)	6 (2.9)
Prosthetic Native Mitral Valve	0 (0)	0 (0)	0 (0)
Native Pulmonary Valve	1 (0.3)	0 (0)	1 (0.5)
Prosthetic Pulmonary Valve	0 (0)	0 (0)	0 (0)
Native Aortic Valve	5 (1.3)	1 (0.6)	4 (1.9)
Prosthetic Aortic Valve	1 (0.3)	0 (0)	1 (0.5)

© 2022 Weis S et al. *JAMA Network Open.*

Other	4 (1)	3 (1.7)	1 (0.5)
**Intra-abdominal Infection**	17 (4.4)	7 (4)	10 (4.8)
Gall bladder, bile ducts	5 (1.3)	2 (1.1)	3 (1.4)
Liver	0 (0)	0 (0)	0 (0)
Pancreas	2 (0.5)	1 (0.6)	1 (0.5)
Peritonitis	9 (2.3)	4 (2.3)	5 (2.4)
**Polymicrobial Bacteremia**	14 (3.6)	6 (3.4)	8 (3.8)
Escherichia coli	5 (1.3)	2 (1.1)	3 (1.4)
Klebsiella pneumoniae	1 (0.3)	1 (0.6)	0 (0)
Enterococcus faecium	0 (0)	0 (0)	0 (0)
Enterococcus faecalis	1 (0.3)	0 (0)	1 (0.5)
Other	6 (1.6)	2 (1.1)	4 (1.9)
**Chronic Disease Category**			
Cardiovascular disease	141 (36.5)	60 (33.9)	81 (38.8)
Respiratory disease	70 (18.1)	31 (17.5)	39 (18.7)
Hepatic disease	33 (8.5)	22 (12.4)	11 (5.3)
Renal disease	50 (13)	25 (14.1)	25 (12)
Immunosuppression	54 (14)	18 (10.2)	36 (17.2)
Diabetes (all)	182 (47.2)	84 (47.5)	98 (46.9)
Cancer/ Leukemia	75 (19.4)	32 (18.1)	43 (20.6)

© 2022 Weis S et al. *JAMA Network Open.*

eTable 3. Additional Patient Characteristics at the Cluster Level

**Characteristics**	**IDC group**, mean [S.D.]	**Control group**, mean [S.D.]
**Focus**		
**Intrathoracic**	24.68 [23.52]	15.01 [12.28]
Bronchitis	2.81 [4.05]	2.5 [5.00]
Pneumonia	19.54 [24.35]	11.98 [10.51]
Mediastinitis	0.76 [2.14]	0 [0]
Empyema	1.86 [6.18]	0.54 [2.21]
Abscess	0 [0]	0 [0]
Other	1.18 [3.52]	0 [0]
**Urogenital**	17.59 [22.89]	15.28 [11.83]
Urinary tract	17.59 [22.89]	14.5 [12.40]
Reproductive system	0 [0]	0.29 [1.21]
Other	0 [0]	0.79 [2.23]
**CNS**	0.74 [3.03]	1.21 [2.69]
Meningitis	0.36 [1.50]	0.42 [1.72]
Abscess	0.74 [3.03]	0.79 [2.23]
Other	0 [0]	0 [0]
**Bone/joint**	15.01 [14.85]	24.08 [15.03]
Spondylodiscitis	6.79 [9.57]	5.22 [6.14]
Osteomyelitis	4.76 [8.10]	7.59 [8.83]
Joint	3.45 [5.22]	8.32 [11.85]
Hip	0.39 [1.62]	0 [0]
Prosthetic hip	0.39 [1.62]	1.18 [4.85]
Knee	0.76 [2.14]	0.49 [2.01]
Prosthetic knee	1.13 [3.35]	4.71 [6.70]
Spine implant	0 [0]	0 [0]
Other	2.35 [5.24]	5.49 [8.31]
**Cardiovascular System**	2.48 [4.15]	6.8 [8.92]
Endocarditis	1.18 [2.62]	6.26 [7.81]
Native Tricuspid Valve	0.42 [1.72]	2.11 [3.97]
Prosthetic Tricuspid Valve	0 [0]	0 [0]
Native Mitral Valve	0.36 [1.5]	3.04 [5.6]
Prosthetic Native Mitral Valve	0 [0]	0 [0]
Native Pulmonary Valve	0 [0]	0.59 [2.43]
Prosthetic Pulmonary Valve	0 [0]	0 [0]
Native Aortic Valve	0.39 [1.62]	1.71 [3.99]
Prosthetic Aortic Valve	0 [0]	0.59 [2.43]
Other	1.29 [2.93]	0.54 [2.21]

© 2022 Weis S et al. *JAMA Network Open.*

**Intra-abdominal Infection**	2.8 [5.3]	4.2 [6.9]
Gall bladder, bile ducts	0.8 [2.2]	1.6 [3.5]
Liver	0 [0]	0 [0]
Pancreas	0.4 [1.6]	0.4 [1.6]
Peritonitis	1.6 [3.9]	2.1 [3.4]
**Polymicrobial Bacteremia**	2.7 [4.5]	3.1 [4.5]
Escherichia coli	1.1 [3.1]	1.1 [2.5]
Klebsiella pneumoniae	0.7 [2.7]	0 [0]
Enterococcus faecium	0 [0]	0 [0]
Enterococcus faecalis	0 [0]	0.5 [2.2]
Other	0.8 [2.3]	1.5 [3.6]
**Chronic Disease Category**		
Cardiovascular disease	33.89 [24.05]	39.49 [12.77]
Respiratory disease	17.72 [14.37]	19.39 [13.95]
Hepatic disease	10.13 [9.11]	5.34 [6.45]
Renal disease	14.02 [9.96]	11.04 [8.97]
Immunosuppression	8.84 [11.56]	18.5 [11.65]
Diabetes (all)	40.76 [22.66]	48.04 [18.72]
Cancer/Leukemia	21.26 [22.21]	19.1 [11.49]

© 2022 Weis S et al. *JAMA Network Open.*

eTable 4. Treatment-Related Adverse Events Reported at the Patient Level (Not Cluster Adjusted)

**Adverse Event**	**Control group (n= 209)**, N (%)	**IDC group (n= 177)**, N (%)
**All**	49 (23,4 %)	26 (14.7 %)
Allergic reaction to antibiotic therapy	9	5
Clostridioides difficile enteritis	5	5
Gastrointestinal bleeding	6	1
Pneumothorax during catheter-replacement	2	2
Liver failure after flucloxacillin	2	1
Liver failure after rifampin	1	0
Renal failure after vancomycin	0	1
Renal failure after aminoglycoside	1	0
Other acute renal failure	5	0
Death during focus sanitation	0	1
Allergy to contrast agent	0	0
Esophageal rupture during transesophageal echocardiography	0	0
Others	18	10

© 2022 Weis S et al. *JAMA Network Open.*

eTable 5. Exploratory Analysis of the Quality-of-Care Indicators Depending on the Order of Study Phases
(A) All clusters and (B) clusters without ID services. * Missing at the patient level.
Abbreviations: RQI.. relative QI improvement, TEE.. transesophageal echocardiography, TTE.. transthoracic echocardiography

A

**Control→IDC (all centers)**					
**Endpoint**	**Missing***	**Control**	**IDC**	**RQI [95 % CI]**	**p-value**
**QI 1: Follow-up blood culture**	0	58 %	85 %	0.46 [0.19; 0.90]	0.02
**QI 2: Focus sanitation**	0	93.9 %	85.7 %	-0.09 [-0.41; 0.12]	0.98
**QI 3: TEE/TTE**	0	55.2 %	87.2 %	0.58 [0.20; 1.34]	0.02
**QI 4: Adequate antibiotic Therapy**	0	49.6 %	83.4 %	0.68 [0.22; 1.88]	0.02
**QI 5: Vancomycin dose adjustment**	0	4.9 %	2.6 %	-0.47 [-1.00; Inf]	0.57
**QI 6: Adequate duration of antibiotic therapy**	7	22.1 %	4.5 %	-0.80 [-1.00; -0.61]	0.16
**Overall QI score (mean points)**	0	283.7	347.6	0.23 [0.01; 0.39]	0.03

**IDC → Control (all centers)**					
**Endpoint**	**Missing***	**Control**	**IDC**	**RQI [95 % CI]**	**p-value**
**QI 1: Follow-up blood culture**	1	74.2 %	88.1 %	0.19 [0.03; 0.50]	0.12
**QI 2: Focus sanitation**	0	93.8 %	97.9 %	0.04 [0; 0.1]	0.15
**QI 3: TEE/TTE**	0	76.5 %	79.4 %	0.04 [-0.04; 0.13]	0.66
**QI 4: Adequate antibiotic Therapy**	0	69.3 %	83.1 %	0.20 [0.01; 0.58]	0.17
**QI 5: Vancomycin dose adjustment**	0	6.7 %	9.5 %	0.42 [-0.41; 2.54]	0.38
**QI 6: Adequate duration of antibiosis**	38	13.6 %	16.2 %	0.19 [-0.48; 1.85]	0.68
**Overall QI score (mean points)**	0	331.2	371.6	0.12 [0.04; 0.24]	0.12

B

**Control→ IDC (w/o centers with ID services)**					
**Endpoint**	**Missing***	**Control**	**IDC**	**RQI [95 % CI]**	**p-value**
**QI 1: Follow-up blood culture**	0	58 %	85 %	0.46 [0.19; 0.93]	0.02
**QI 2: Focus sanitation**	0	93.9 %	85.7 %	-0.09 [-0.41; 0.12]	0.98
**QI 3: TEE/TTE**	0	55.2 %	87.2 %	0.58 [0.22; 1.34]	0.02
**QI 4: Adequate antibiotic Therapy**	0	49.6 %	83.4 %	0.68 [0.22; 1.88]	0.02

© 2022 Weis S et al. *JAMA Network Open.*

**QI 5: Vancomycin dose adjustment**	0	4.9 %	2.6 %	-0.47 [-1.00; Inf]	0.57
**QI 6: Adequate duration of antibiosis**	7	22.1 %	4.5 %	-0.80 [-1.00; -0.60]	0.16
**Overall QI score (mean points)**	0	283.7	347.6	0.22 [0.10; 0.39]	0.04

**IDC → Control (w/o centers with ID services)**					
**Endpoint**	**Missing***	**Control**	**IDC**	**RQI [95 % CI]**	**p-value**
**QI 1: Follow-up blood culture**	1	69 %	86.8 %	0.26 % [0.07; 0.69]	0.06
**QI 2: Focus sanitation**	0	95 %	99.2 %	0.04 [0; 0.11]	0.07
**QI 3: TEE/TTE**	0	73.3 %	77.7 %	0.06 [-0.03; 0.16]	0.56
**QI 4: Adequate antibiotic Therapy**	0	61.6 %	81.5 %	0.32 [0.07; 0.84]	0.06
**QI 5: Vancomycin dose adjustment**	0	6.2 %	8.4 %	0.34 [-0.71; 4.09]	0.56
**QI 6: Adequate duration of antibiosis**	30	14.7 %	17.3 %	0.18 [-0.53; 2.29]	0.72
**Overall QI score (mean points)**	0	316.5	367.8	0.16 [0.06; 0.31]	0.08

eFigure. Exploratory Analysis of 18-Month Survival With Kaplan-Meier Plots for Individual Participants From (A) All Centers, (B) Centers Without Infectious Disease Consultation (IDC), and (C) for the Composite (Survival and No Readmission) for All Centers

Abbreviations: IDC.. infectious disease consultation.

A

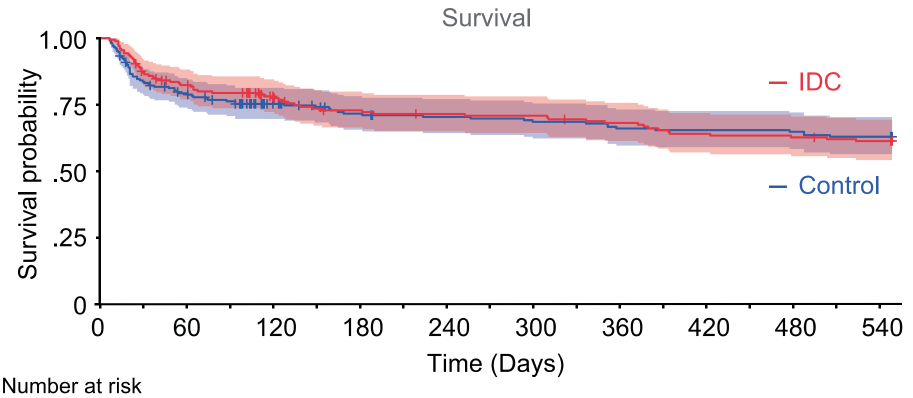

Number at risk

IDC	177	140	120	109	106	105	100	94	92	89
Control	209	161	131	117	113	110	106	105	104	101

B

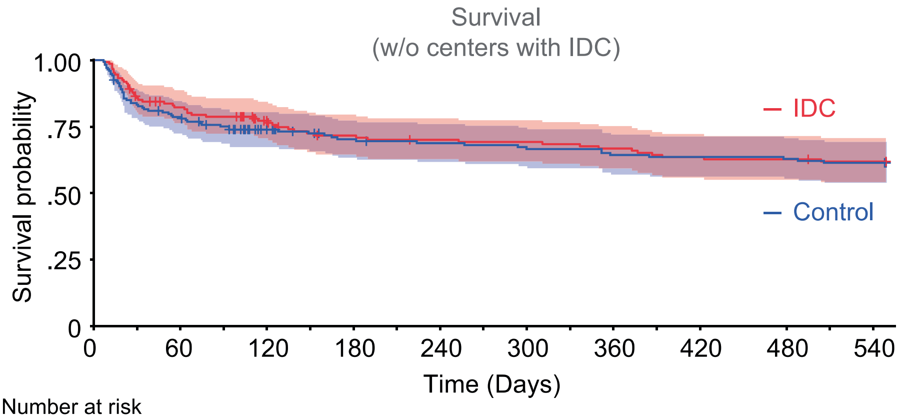

Number at risk

IDC	148	116	99	89	86	85	82	78	77	75
Control	174	135	107	96	93	90	87	86	85	83

C

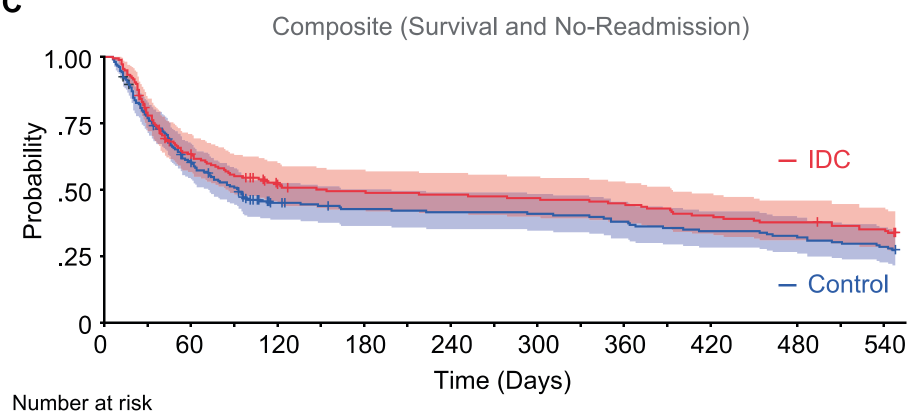

Number at risk

IDC	177	108	83	76	74	72	69	62	58	53
Control	209	123	79	72	70	69	64	58	55	48

© 2022 Weis S et al. *JAMA Network Open.*
