# Supplementary material for: Effect of Automated Telephone Infectious Disease Consultations to Nonacademic Hospitals on 30-Day Mortality Among Patients With Staphylococcus aureus Bacteremia: The SUPPORT Cluster Randomized Clinical Trial
Source: JAMA Netw Open. 2022 Jun 24;5(6):e2218515. doi: 10.1001/jamanetworkopen.2022.18515 (PMC9233240; doi:10.1001/jamanetworkopen.2022.18515)
Supplement: Supplement 3. — Nonauthor Collaborators [file jamanetwopen-e2218515-s003.pdf]

Supplemental Online Content: Nonauthor Collaborators
*First name, last name, and suffix (if applicable) are required and will appear in PubMed.

*Group Name(s): The SUPPORT Study Group							
***First Name and Middle Initial(s)**	***Last Name**	***Suffix (eg, Jr, III)**	Academic Degrees	Institution	Location (city, state/province, country)	Role or Contribution, eg, chair, principal investigator	Group (if more than 1 Group listed in the byline) and/or Subgroup (eg, Steering Committee)
Uwe	Schotte		Dr.med.	Eichsfeld Klinikum Worbis, Reifenstein, Heiligenstadt;	Worbis, Reifenstein, Heiligenstadt; Germany	local PI	
Attila	Yilmaz		Prof.Dr.	Elisabeth Klinikum Schmalkalden	Schmalkalden, Germany	local PI	
Claudia	Höpner		Dr.med.	Helios Klinikum Erfurt	Erfurt, Germany	local PI	
Volkmar	Schenk		MD	Helios Klinikum Gotha	Gotha, Germany	local PI	
Margarete	Borg-von Zepelin		Prof.Dr	Hufeland Klinikum Mühlhausen/Bad Langensalza	Hufeland Klinikum Mühlhausen/Bad Langensalza, Germany	local PI	
Marc	Morgenfrüh		MD	Ilm-Kreis-Kliniken Arnstadt/Ilmenau	Arnstadt/Ilmenau, German	local PI	
Roland	Göb		Dr.med.	Katholisches Krankenhaus St. Johann Nepomuk Erfurt	Erfurt, Germany	local PI	
Anagnsotina	Orfanou		MD	Klinikum Altenburg	Altenburg, Germany	local PI	
Gottschalk	Peter		Dr.med.	Kreiskrankenhaus Greiz	Greiz, Germany	local PI	
Holger	Rupprecht		Dipl.med	Kreiskrankenhaus Schleiz	Schleiz, Germany	local PI	
Christof	Lascho		Dr.med.	Sophien- und Hufeland Klinikum Weimar	Weimar, Germany	local PI	
Christian	Schmidt		Dr.med.	SRH Klinikum Gera	Gera, Germany	local PI	
Martina	Paul		Dipl.med	SRH Klinikum Gera	Gera, Germany	local PI	
Alexander	Spengler		MD	SRH Klinikum Suhl	Sulh, Germany	local PI	
Karsten	Kromann		Dr.med.	Südharz Klinikum Nordhausen	Nordhausen, Germany	local PI	
Jörg	Epstude		Dr.med.	Thüringen-Kliniken “Georgius Agricola”	Pößneck, Rudolstadt, Saalfeld, Germany	local PI	
