# Supplementary material for: Effect of Automated Telephone Infectious Disease Consultations to Nonacademic Hospitals on 30-Day Mortality Among Patients With Staphylococcus aureus Bacteremia: The SUPPORT Cluster Randomized Clinical Trial
Source: JAMA Netw Open. 2022 Jun 24;5(6):e2218515. doi: 10.1001/jamanetworkopen.2022.18515 (PMC9233240; doi:10.1001/jamanetworkopen.2022.18515)
Supplement: Supplement 4. — Data Sharing Statement [file jamanetwopen-e2218515-s00.pdf]

Data Sharing Statement

Weis. Effect of Automated Telephone Infectious Disease Consultations to Nonacademic Hospitals on 30-Day Mortality Among Patients With Staphylococcus aureus Bacteremia. *JAMA Netw Open*. Published June 24, 2022. doi:10.1001/jamanetworkopen.2022.18515

Data
Data available: Yes
Data types: Data dictionary
How to access data: Data dictionary will be avaialable upon reasonable request from the first author
When available: With publication

Supporting Documents
Document types: None

Additional Information
Who can access the data: n/a
Types of analyses: n/a
Mechanisms of data availability: n/a
Any additional restrictions: n/a
